# Crystalline–amorphous homojunction engineering in carbon nitride for solar-driven nitrate into ammonia conversion

**DOI:** 10.1039/d6sc01863g

**Published:** 2026-05-29

**Authors:** Yiyang Chen, Yuxiang Zhu, Xiang Zhong, Yuhang Liang, Qiufan Sun, Sai Xu, Jianfeng Yao

**Affiliations:** a Jiangsu Co-Innovation Center of Efficient Processing and Utilization of Forest Resources, College of Chemical Engineering, Nanjing Forestry University Nanjing Jiangsu 210037 China yxzhu89@njfu.edu.cn jfyao@njfu.edu.cn; b College of Chemistry, Fuzhou University Fuzhou Fujian 350108 China; c School of Physics, The University of Sydney New South Wales 2006 Australia; d Jiangsu Key Laboratory of Chemical Pollution Control and Resources Reuse, School of Environmental and Biological Engineering, Nanjing University of Science and Technology Nanjing Jiangsu 210094 China

## Abstract

Photocatalytic nitrate to ammonia conversion offers a promising route to close the nitrogen cycle, yet is limited by inefficient charge separation and poor reaction selectivity. Here, crystalline-phase-engineered oxygen-doped carbon nitride homojunctions are constructed by integrating poly(heptazine–triazine) imides (PHTI), poly(heptazine imide) (PHI) and poly(triazine imide) (PTI) into oxygen-doped carbon nitride microspheres (OCNMs), forming S-scheme, type-II and type-I architectures. Among them, the urchin-like S-scheme PHTI/OCNMs homojunction achieves an ammonia formation rate of 893 µmol g_cat_^−1^ h^−1^ with an apparent quantum efficiency of 6.28% at 400 nm, far exceeding its type-II and type-I analogues. *In situ* probe techniques and theoretical calculations demonstrate that a strong built-in electric field enforces S-scheme charge separation, retaining high-energy electrons on OCNMs for nitrate reduction and high-energy holes on PHTI for oxidation. The synergistic effects of crystalline phase alignment, oxygen-doped sites, and S-scheme interfacial recombination enhance nitrate adsorption, stabilize *HNO intermediates and lower the free-energy barriers toward *NH_3_, enabling NH_4_^+^ selectivity above 94%. The PHTI/OCNMs catalyst remains active under unconcentrated natural sunlight and in real water matrices, highlighting the practical potential of crystalline homojunction engineering for solar-driven nitrate valorization.

## Abstract 2

Crystalline–amorphous carbon nitride homojunctions (S-scheme, type-II and type-I) were fabricated from PHTI, PHI, PTI, and OCNMs. The urchin-like S-scheme PHTI/OCNMs exhibits the highest nitrate to ammonia conversion with >94% NH_4_^+^ selectivity.

## Graphical abstract

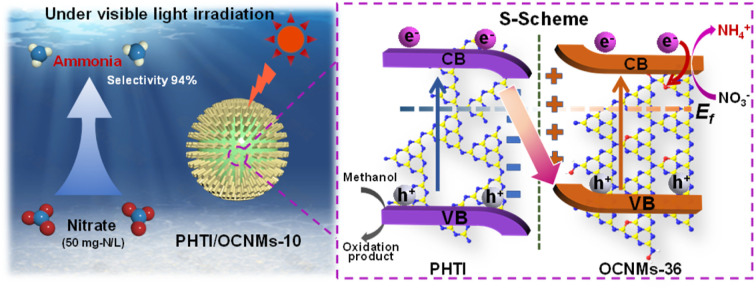

## 1. Introduction

Ammonia (NH_3_) serves not only as a vital chemical feedstock extensively employed in the agricultural and chemical industries, but also as a promising hydrogen carrier within the framework of sustainable energy systems.^1^ Currently, the large-scale synthesis of NH_3_ is primarily achieved *via* the Haber–Bosch process, which necessitates harsh reaction conditions, to facilitate the direct conversion of nitrogen (N_2_) and hydrogen (H_2_) gases. Although highly productive, this energy-intensive process contributes significantly to anthropogenic carbon dioxide emissions.^2,3^ Meanwhile, nitrate (NO_3_^−^), a widespread environmental contaminant predominantly originating from agricultural leaching and industrial effluents, poses significant ecological and health hazards owing to its high aqueous solubility and mobility. Given these considerations, the photocatalytic reduction of NO_3_^−^ to NH_3_ under ambient conditions represents an attractive alternative, offering a low-energy pathway for nitrogen valorization.^4,5^ This approach not only addresses the environmental burden of nitrate pollution but also aligns with circular nitrogen economy principles, providing a dual-function strategy for both pollutant remediation and renewable ammonia production.

Graphitic carbon nitride (CN) has garnered attention for its suitable band gap, layered structure, and earth-abundant precursors. However, its high exciton binding energy hampers charge separation, whereas solid-state synthesis often yields defect-rich amorphous phases that facilitate charge recombination and reduce photocatalytic performance.^6^ A variety of modification strategies, such as morphology design and homojunction construction, have thus been extensively explored and developed.^7^ Three-dimensional carbon nitride microspheres (CNMs) *via* hydrothermal synthesis are of considerable interest in photocatalysis because of their high surface area, structural stability, and suitability for spatial functionalization. Moreover, the compartmentalized architecture enables precise heterojunction design, promoting efficient charge separation and enhanced photo-redox performance.^8,9^

Increased crystallinity of CN facilitates the transport of charge carriers by reducing the number of defect-induced recombination centers, while simultaneously extending the π-conjugated system. Consequently, extensive research efforts have been directed toward the synthesis and application of crystalline carbon nitrides (CCNs). Poly(heptazine imide) (PHI), poly(triazine imide) (PTI), and poly(heptazine–triazine) imides (PHTI) are structural derivatives of CCNs, distinguished by variations in their framework composition and connectivity arising during the formation process.^10–12^ PHI features a crystalline architecture composed of six heptazine units arranged in a hexagonal configuration and bridged by imide linkages.^13^ In contrast, PTI, which is typically less thermally stable than PHI, adopts a triazine-based framework. PHTI represents a hybrid architecture that integrates both PHI and PTI motifs, combining their structural features and potentially synergizing their photocatalytic properties.^14^ These CCNs have demonstrated considerable potential in various photocatalytic applications, including hydrogen evolution,^15^ ammonia synthesis,^16^ and nitrogen oxide decomposition.^17^

The deliberate construction of homojunction architectures offers a versatile and effective strategy for photocatalysis. Homojunctions formed within a single semiconductor, yet differing in crystal phase, morphology, or electronic structure, benefit from strong interfacial coupling and excellent lattice compatibility, thereby reducing charge carrier migration barriers and promoting efficient separation.^18,19^ When coupled with well-matched band alignments, such architectures provide multiple active sites for charge generation and diverse transport pathways, leading to enhanced light harvesting, suppressed recombination, improved photostability, and elevated overall photocatalytic performance.^20^ For instance, PHI crystallites were epitaxially grown on PTI layers *via* a molten salt method to construct a PHI/PTI homojunction, which promoted directional charge transfer from PTI to PHI and generated an electron-rich interface for efficient oxygen reduction.^21^ CN quantum dots anchored on amorphous CN layers construct an S-scheme homojunction, where matched structure and band alignment promote charge separation and photocatalytic hydrogen evolution. In addition, a PHI crystalline–amorphous junction constructed through fragmentation and directed healing of bulk CN significantly enhanced charge-transfer dynamics and spatial separation of redox centers. Owing to the feasibility of the molten salt synthesis approach, PTI, PHI, and PHTI can be selectively integrated onto CNMs to construct homojunctions with crystalline structure regulation, which holds great potential for further enhancing the efficiency of NO_3_^−^-to-NH_3_ conversion under visible-light irradiation.

In this work, oxygen-doped CNMs (OCNMs) were integrated with PHI, PTI and PHTI allotropes, respectively, to construct a series of crystalline homojunction photocatalysts for enhanced nitrate to ammonia conversion. Their structural, surface, and electronic properties were systematically characterized. Interfacial charge transfer behavior was elucidated by *in situ* probe techniques and theoretical calculations, providing mechanistic insight into the S-scheme-driven reaction pathway. The nitrate reduction activity was further evaluated in diverse water matrices and under natural solar irradiation. This work highlights crystalline-phase engineering as an effective strategy for solar ammonia synthesis.

## 2. Results and discussion

### 2.1 Structural characterization

**Fig. 1:**
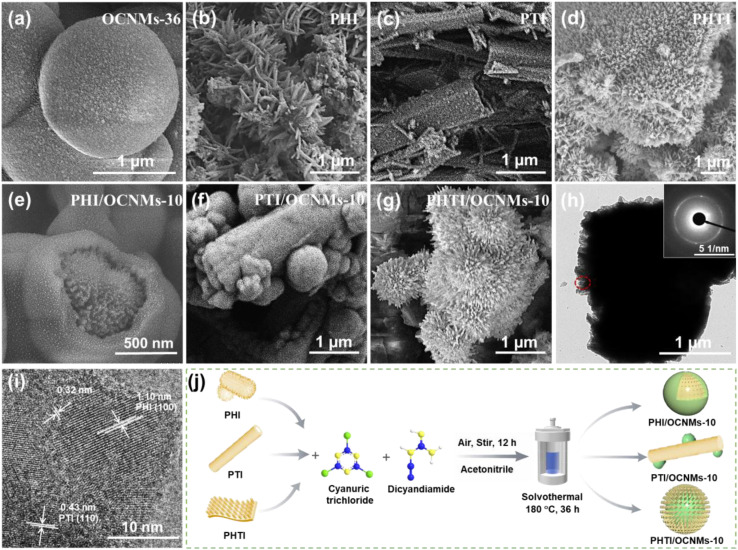
SEM images of OCNMs-36 (a), PHI (b), PTI (c), PHTI (d), PHI/OCNMs-10 (e), PTI/OCNMs-10 (f) and PHTI/OCNMs-10 (g), and TEM images (h and i) of PHTI/OCNMs-10. Illustration of the fabrication process of the PHI/OCNMs-10, PTI/OCNMs-10 and PHTI/OCNMs-10 (j).

Scanning electron microscopy (SEM) and transmission electron microscopy (TEM) analyses reveal that OCNMs-36 possesses a highly uniform microspherical morphology, with diameters ranging from approximately 1 to 3 µm. These microspheres exhibit a solid internal structure accompanied by a characteristically rough surface texture (Fig. 1a and S2a, b). The overall morphology of the OCNMs-*x* (*x* = 24, 30, 36, 42 and 48 h) microspheres remains consistent across different synthesis durations (Fig. S2c–f), despite notable variations in crystallinity and oxygen doping, as further analyzed by the SEM energy-dispersive X-ray (EDX) and X-ray diffraction (XRD) analyses. PHI (Fig. 1b) consists of well-defined, one-dimensional nanorods grown on nanoparticles or nanorods, while the PTI material (Fig. 1c) presents a non-uniform tubular structure with tube diameters ranging from several tens of nanometers to approximately one micrometer. These morphological differences arise from the variation in the precursor materials used during the molten salt synthesis process. For PHTI, vertically aligned, needle-like nanorods, tens of nanometers in diameter and less than 1 µm in length, are uniformly grown on the surface of the nanosheets (Fig. 1d).

The PHI/OCNMs-10 composite displays a distinct core–shell structured morphology, characterized by the radial growth of nanorods within the interior of the spheres (Fig. 1e and S2g). Moreover, a pronounced interfacial discontinuity is evident between the needle-like PHI and the surrounding microspherical shell of OCNMs-36. In the case of PTI/OCNMs-10, a hierarchical architecture is observed, wherein tubular structures are decorated with spherical particles on their external surfaces (Fig. 1f and S2h). By contrast, the PHTI/OCNMs-10 composite exhibits a hierarchical three-dimensional (3D) urchin-like architecture, characterized by a well-ordered array of nanorods protruding from the exterior surfaces of the microspheres (Fig. 1g and h). This structural arrangement is probably facilitated by the presence of nanosheets within the PHTI matrix, which serve as anchoring sites for outward-oriented nanorod growth. The distinct diffraction rings observed in the black-circled region of Fig. 1h inset confirm the high crystallinity of the surface nanorods in PHTI/OCNMs-10. High-resolution TEM image (Fig. 1i) of PHTI/OCNMs-10 reveals distinct lattice fringes with spacings of 1.10 and 0.43 nm, corresponding to the (100) plane of PHI and (110) plane of PTI, respectively.^22,23^ Additional fringes of 0.32 nm are assigned to the (002) planes of heptazine or triazine units, further demonstrating well-defined crystallinity.^24^ The well-defined lattice fringes provide direct evidence for the successful *in situ* formation of the PHTI on OCNMs. Additionally, SEM-EDX elemental mapping (Fig. S2j and S3) confirms the homogeneous distribution of C, N and O elements in both OCNMs-*x* and PHTI/OCNMs-10. Quantitative elemental contents were further determined by elemental analysis (Table S1). Notably, the oxygen doping content exhibits a time-dependent evolution with increasing solvothermal treatment duration, reaching a maximum at 36 h before declining at extended durations. This trend suggests a dynamic equilibrium in oxygen incorporation. Furthermore, elemental analysis-derived N/O atomic ratios corroborate this observation, providing additional evidence for the variation in oxygen content with treatment time.

**Fig. 2:**
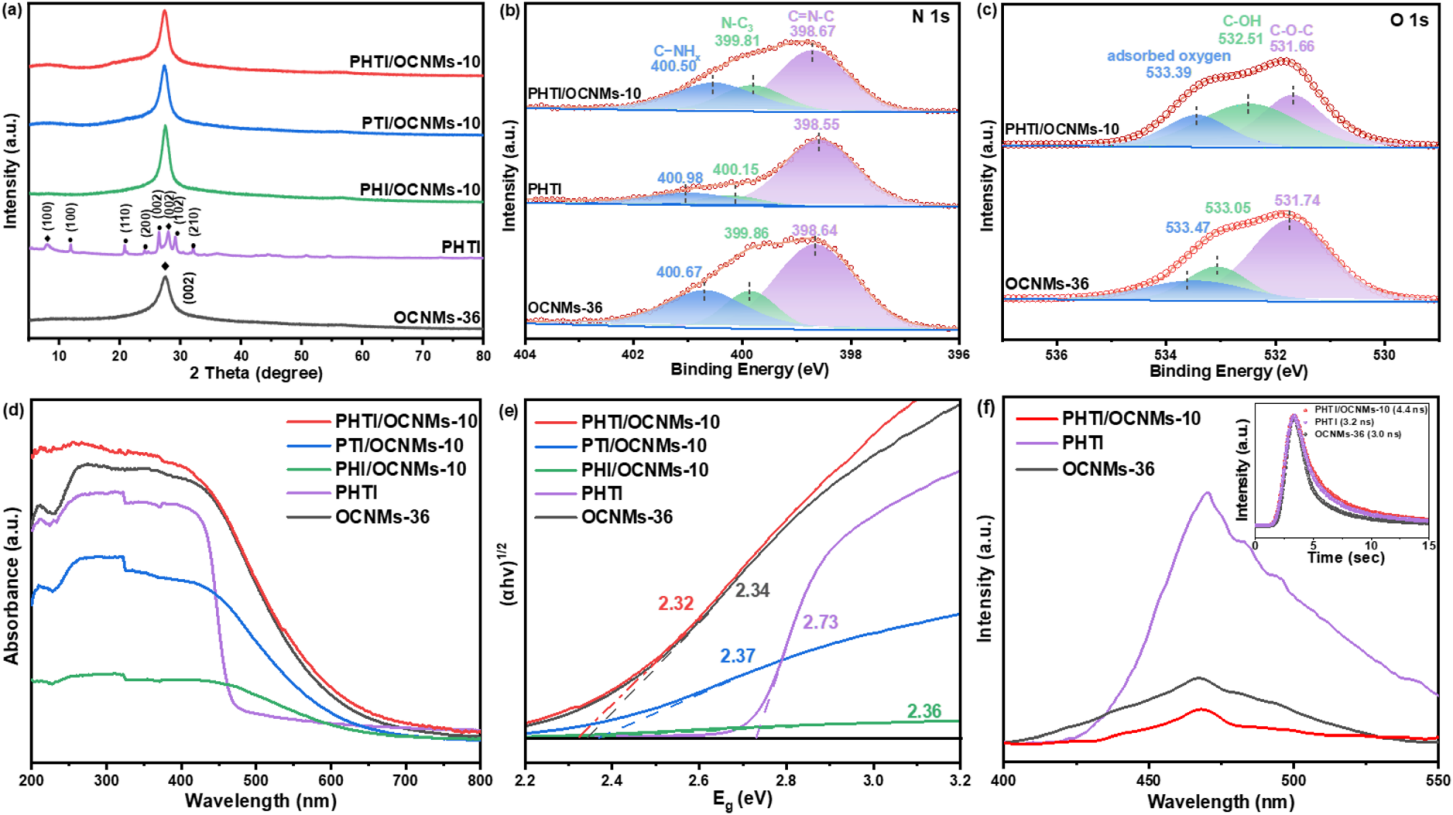
XRD patterns (a) of the OCNMs-36, PHTI, PHI/OCNMs-10, PTI/OCNMs-10 and PHTI/OCNMs-10 catalysts. High-resolution N 1s (b) and O 1s (c) X-ray photoelectron spectroscopy (XPS) spectra of OCNMs-36, PHTI and PHTI/OCNMs-10. UV-vis DRS spectra (d) and associated Kubelka–Munk transformations (e) of the various catalysts. The steady-state and time-resolved PL spectra (f) of OCNMs-36, PHTI and PHTI/OCNMs-10.

The XRD diffraction pattern of OCNMs-36 displays a broad peak at 27.5°, characteristic of graphitic interlayer stacking (Fig. 2a).^25^ With increasing solvothermal time, the progressive narrowing of this peak indicates enhanced crystallinity, while its slight redshift suggests enlarged interlayer spacing (Fig. S4a).^26^ PHTI exhibits diffraction features from both PHI and PTI phases: peaks at 8.1° and 28.0° correspond to the (100) and (002) planes of PHI, whereas reflections at 12.0°, 20.8°, 24.1°, 26.4°, 29.2°, and 32.2° are characteristic of PTI (Fig. S4b), confirming a copolymeric PHI-PTI structure.^27,28^ In the composites, only the (002) peak of OCNMs-36 is observed (Fig. 2a and S4c), indicating preservation of the layered framework and the low loading of crystalline PHTI. Fourier transform infrared spectroscopy (FTIR) spectrum of OCNMs-36 displays bands at *ca.* 3700–3000, 1700–1200, and 810 cm^−1^, corresponding to N–H/O–H stretching, C=N and C–N vibrations in the heptazine framework, and the heptazine breathing mode, respectively (Fig. S4e).^29^ OCNMs-*x* exhibit similar FTIR spectra, indicating comparable structural features. PTI, PHI, and PHTI show similar profiles, with PHTI exhibiting an additional –C≡N band at ∼2175 cm^−1^ (Fig. S4f and g),^30^ indicating structural modification. The composite catalysts retain the characteristic bands of OCNMs-36, confirming framework integrity after coupling. Notably, PHTI/OCNMs-10 exhibits a higher BET surface area and larger pore size than OCNMs-36 (Fig. S5a and b), providing improved exposure of active sites.

Survey XPS spectra show the existence of C, N, and O elements in the respective samples (Fig. S5c). Since potassium was not detected in the three homojunction composites, it was excluded from comparative analysis. The high-resolution C 1s XPS spectrum of OCNMs-36 (Fig. S5d) can be resolved into three components at binding energies of *ca.* 288.77, 286.42, and 284.83 eV, which are attributed to carbon atoms in the N–C=N, C–O/C–NH_*x*_, and C–C components, respectively.^31^ Correspondingly, the N 1s spectrum of OCNMs-36 (Fig. 2b) reveals three characteristic peaks at 400.67, 399.86, and 398.64 eV, assigned to C–NH_*x*_, N–C_3_ and C=N–C species, respectively.^32^ The deconvoluted O 1s spectrum of OCNMs-36 exhibits three peaks centered at 533.44, 532.76, and 531.57 eV (Fig. 2c), which can be ascribed to adsorbed oxygen species, C–OH, and C–O–C bonds, respectively.^33,34^ It provides strong evidence for the successful incorporation of oxygen atoms within the OCNMs-36 framework. A similar spectral profile is observed for PHTI, suggesting the preservation of key bonding environments. Notably, for the PHTI/OCNMs-10 composite, the binding energies associated with the N–C=N and C–C species in the C 1s spectrum, as well as the C–NH_*x*_, N–C_3_ and C=N–C components in the N 1s region, appear between those of pristine OCNMs-36 and PHTI. This shift in binding energy confirms the formation of a homojunction interface between the two components, indicative of strong interfacial electronic interactions.^25^ In addition, the valence band (VB) XPS spectra of PHI, PTI, PHTI and OCNMs-36 reveal VB edge positions (*E*_VB_) at 1.37, 1.46, 1.83, and 1.40 V *vs.* the normal hydrogen electrode (NHE), respectively (Fig. S5e). Among these, PHTI has the most positive VB edge, suggesting its superior oxidative capability.

### 2.2 Optical and electronic properties

UV-visible diffuse reflectance spectroscopy (UV-vis DRS) was employed to elucidate the light-harvesting properties and electronic band structures of the synthesized catalysts. The OCNMs-36 exhibits absorption in the visible light region, and its optical absorption characteristics are significantly influenced by the duration of the solvothermal treatment. Specifically, with increasing solvothermal time, the absorption edge of OCNMs-*x* shifts from approximately 580 nm to 630 nm, accompanied by a gradual narrowing of the band gap (Fig. S6a and b). This red shift and bandgap reduction can be attributed to enhanced n–π* electronic transitions and a concomitant decrease in crystallinity.^35^ The visible-light absorption behaviour of the composite catalysts, including PHI/OCNMs-10, PTI/OCNMs-10 and PHTI/OCNMs-10, varies substantially, with corresponding absorption band edges located at approximately 650, 638, and 668 nm (Fig. 2d), and estimated bandgap energies (*E*_g_) of *ca.* 2.36, 2.37 and 2.32 eV (Fig. 2e), respectively.^36^ Among these, PHTI/OCNMs-10 displays the most extended absorption edge, indicating that the incorporation of PHTI effectively broadens the photo-responsive spectral range of the resulting homojunction system. Additionally, the bandgap energies of the PHI, PTI, PHTI and pristine OCNMs-36 individual components are estimated to be 2.67, 2.98, 2.73 and 2.34 eV, respectively (Fig. S6c and d). Notably, the *E*_g_ value of PHTI/OCNMs-10 is narrower than those of both bare PHTI and OCNMs-36, suggesting enhanced generation of photoexcited charge carriers under visible light irradiation. Furthermore, varying the PHTI loading in PHTI/CNMs-*y* (*y* = 5, 10, 15, 20) reveals that 0.1 g achieves the most extended absorption edge, enhancing visible-light harvesting. Excessive PHTI, however, diminishes absorption due to surface coverage or aggregation hindering photon penetration (Fig. S6e and f). These results indicate that constructing a homojunction with optimized PHTI content effectively modulates the electronic structure, extending light absorption and boosting visible-light photocatalytic activity.

To gain deeper insight into the photogenerated charge generation and separation behaviours across various samples, steady-state and time-resolved photoluminescence (PL) measurements were performed. Under 330 nm excitation, all samples display a typical emission centered at ∼470 nm (Fig. 2f). Notably, PHTI/OCNMs-10 exhibits the weakest PL intensity, indicating effectively suppressed charge recombination due to the formation of a homojunction with an internal electric field.^37^ Time-resolved PL analysis (inset of Fig. 2f) reveals an extended average carrier lifetime for PHTI/OCNMs-10 (4.4 ns), compared with OCNMs-36 (3.0 ns) and PHTI (3.2 ns), further confirming enhanced charge separation and transfer. Transient photocurrent measurements (Fig. S7a) show that PHTI/OCNMs-10 generates a higher photocurrent density than OCNMs-36, consistent with the PL results and indicative of improved carrier separation and transport. This enhancement is attributed to the favorable electron transport properties of PHTI, which facilitate interfacial charge transfer to the substrate. In addition, the photothermal behavior was evaluated under visible-light irradiation (Fig. S8). While OCNMs-36 exhibits a moderate temperature rise, PHTI/OCNMs-10 shows a markedly enhanced photothermal response, originating from the presence of PHTI and the intimate homojunction interface. The synergistic effect of light-induced photothermal conversion and efficient charge separation therefore contributes significantly to the enhanced photocatalytic performance.

### 2.3 Photocatalytic performance

**Fig. 3:**
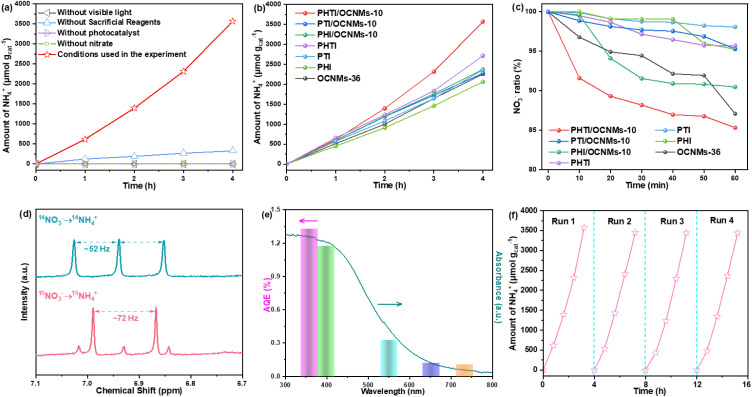
Control experiments of PHTI/OCNMs-10 under different reaction conditions (a). Visible-light ammonia generation over OCNMs-36, PHI, PTI, PHTI, PHI/OCNMs-10, PTI/OCNMs-10 and PHTI/OCNMs-10 (b). Time-resolved adsorption experiments for OCNMs-36, PHI, PTI, PHTI, PHI/OCNMs-10, PTI/OCNMs-10 and PHTI/OCNMs-10 in NO_3_^−^ solution (c). ^1^H NMR spectra of ammonia generated using ^14^NH_4_^+^ and ^15^NH_4_^+^ as nitrogen sources (d). The apparent quantum efficiency (AQE)–wavelength dependence of PHTI/OCNMs-10 in photocatalytic nitrate reduction (e). The cycle experiment of PHTI/OCNMs-10 (f).

The photocatalytic nitrate reduction activity of the as-prepared catalysts was systematically evaluated under visible light irradiation, employing methanol as a hole scavenger and KNO_3_ (50 mg-N L^−1^, equivalent to 222 mg L^−1^ NO_3_^−^) as the nitrogen source. The quantification of ammonia was conducted using Nessler's method (Fig. S9a).^38^ Control experiments were conducted to eliminate environmental interference. Negligible NH_4_^+^ production was observed in the absence of either NO_3_^−^, photocatalyst, or light, confirming their necessity for the photocatalytic process (Fig. 3a). The time-dependent ammonia production was compared across OCNMs-*x* and the activity increases with prolonged hydrothermal treatment, peaking at 36 h, followed by a decline at longer durations. It suggests that optimal catalytic activity correlates with the highest oxygen doping level achieved at the 36 h hydrothermal treatment (Fig. S10a). Comparing the ammonia production of OCNMs-36 at varying nitrate concentrations, the activity increases with nitrate concentration, reaches a maximum at 50 mg-N L^−1^, and subsequently declines (Fig. S10b). After 4 h irradiation, pristine OCNMs-36, PHI, PTI and PHTI catalysts exhibit ammonia generation of 2258, 2062, 2363 and 2714 µmol g_cat_^−1^, respectively (Fig. 3b). By combining PHI, PTI and PHTI onto the OCNMs-36 support, all resulting composites present enhanced NH_4_^+^ production rates compared to pristine OCNMs-36. At a loading content of 10 wt%, the PHTI-decorated OCNMs-36 demonstrates an enhancement in ammonia synthesis activity, achieving an ammonia yield rate of 893 µmol g_cat_^−1^ h^−1^, surpassing those of PHI/OCNMs-10 (593 µmol g_cat_^−1^) and PTI/OCNMs-10 (569 µmol g_cat_^−1^) under identical conditions. The improved activity could be primarily attributed to the 3D urchin-like structure, which enhances light harvesting and active sites, along with the closely integrated homojunction that facilitates efficient charge separation. Furthermore, a positive correlation between the PHTI loading and photocatalytic activity was observed up to this optimal loading. However, further increases in the PHTI content beyond 10 wt% lead to a decline in NH_4_^+^ yield, likely due to PHTI nanorod aggregation, which diminishes the active site availability and overall utilization efficiency (Fig. S10c).

In addition to NH_3_, potential by-products including nitrite (NO_2_^−^), N_2_ and H_2_ were quantified over the various photocatalysts using UV-vis spectroscopy and gas chromatography (Fig. S9b–e). Notably, no detectable H_2_ was observed in any of the reaction systems, indicating the high selectivity of these catalysts toward nitrogenous products. Among the intermediates, NO_2_^−^ was identified as the primary detectable species; however, its concentration remains low across all the systems (Fig. S10d), likely due to its rapid consumption during the reduction pathway.^39^ Remarkably, among the tested materials, the PHTI/OCNMs-10 composite exhibits the lowest N_2_ yield, which may be attributed to the strong surface adsorption of nitroxyl (HNO) or its rapid reduction facilitated by photogenerated electrons in the effective homojunction.^40^ For the PHTI/OCNMs-10 photocatalyst, the nitrate conversion efficiency reaches 73% at an initial NO_3_^−^ concentration of 50 mg-N L^−1^. Among the reduction products, NH_4_^+^ is the predominant species with a high selectivity of 94.62%, while the selectivity for N_2_ and NO_2_^−^ is significantly lower, at 5.36% and 0.02%, respectively. The high NH_4_^+^ selectivity underscores the potential of PHTI/OCNMs-10 as a highly effective photocatalyst for nitrogen-cycle applications.

The adsorption behaviors of NO_3_^−^ and NH_4_^+^ on OCNMs-36, PHI, PTI, PHTI and PHTI/OCNMs-10 onto the CNM were evaluated to elucidate their surface interaction capabilities. Although OCNMs-36 possesses a lower surface area than PHTI, its higher nitrate adsorption highlights the critical role of oxygen doping in enhancing NO_3_^−^ affinity. Moreover, the NO_3_^−^ adsorption capacity of the PHTI/OCNMs-10 composite was measured to be 17 µmol g_cat_^−1^, which is greater than that of its other counterparts (Fig. 3c). This enhancement is likely attributable to the synergistic effect of the nanorod and microsphere architecture and doped oxygen atoms in PHTI/OCNMs-10, which facilitates additional NO_3_^−^ enrichment through physical confinement or capillary condensation. In contrast, the NH_4_^+^ adsorption performance (Fig. S10e) exhibits negligible uptake across the examined photocatalysts. This indicates a weak interaction between the catalysts and NH_4_^+^, facilitating their rapid desorption and thereby potentially promoting the efficient photocatalytic formation of NH_4_^+^. To identify the nitrogen source of the generated NH_3_, isotopic labeling experiments were conducted under visible light irradiation for 8 h. As shown in Fig. 3d, characteristic triplet and doublet peaks corresponding to ^14^NH_4_^+^ and ^15^NH_4_^+^, respectively, were observed in the ^1^H NMR spectra when ^14^NO_3_^−^ and ^15^NO_3_^−^ were employed as nitrogen sources.^41^ This clearly confirms that the NO_3_^−^ feedstock is indeed reduced to NH_4_^+^ during the reaction. The ammonia generated after 8 h of reaction was quantified *via* both NMR and Nessler's reagent methods (Fig. S11a–c), which yielded consistent results, thereby validating the accuracy and reliability of the quantification.

PHTI/OCNMs-10 exhibits a wavelength-dependent AQE, decreasing from 7.10% at 360 nm to 0.58% at 730 nm, with a notable 6.28% at 400 nm (Fig. 3e), comparable to state-of-the-art CN-based photocatalysts (Table S2). The resulting AQE values exhibit a pronounced dependence on excitation wavelength, which closely aligns with the optical absorption profile of PHTI/OCNMs-10. This correlation indicates efficient photon-to-charge carrier conversion across the examined spectral range, thereby validating the effectiveness of PHTI/OCNMs-10 in harvesting and utilizing incident photons for photocatalytic processes. The effects of various nitrate salts were further evaluated, showing nearly identical reduction performance and thus negligible influence on the reaction process (Fig. S11d). Sacrificial reagents play a crucial role in optimizing photocatalytic nitrate reduction to NH_3_. Comparative results indicate that methanol delivers the highest activity among the tested electron donors (Fig. S11e), attributed to its efficient hole-scavenging ability that suppresses electron–hole recombination. The structural integrity and operational stability of catalyst were further evaluated. After four consecutive photocatalytic cycles, the NH_3_ production exhibits only a marginal decrease of 3.6% relative to the initial cycle (Fig. 3f), indicating remarkable catalytic durability. Furthermore, the characterization results reveal that the morphology (Fig. S2i), crystalline structure (Fig. S4d), and chemical composition (Fig. S4h) of PHTI/OCNMs-10 remain largely unaltered, demonstrating the excellent structural integrity and chemical stability under prolonged photocatalytic conditions. Moreover, the catalyst demonstrates good long-term stability, maintaining an ammonia generation rate over 500 µmol g_cat_^−1^ h^−1^ for the last cycle over 32 h (Fig. S11f). In addition, the PHTI/OCNMs-10 catalyst enables N_2_ photoreduction to NH_3_, albeit with a significantly lower yield than that from NO_3_^−^ (Fig. S11g).

### 2.4 Mechanism for ammonia formation

**Fig. 4:**
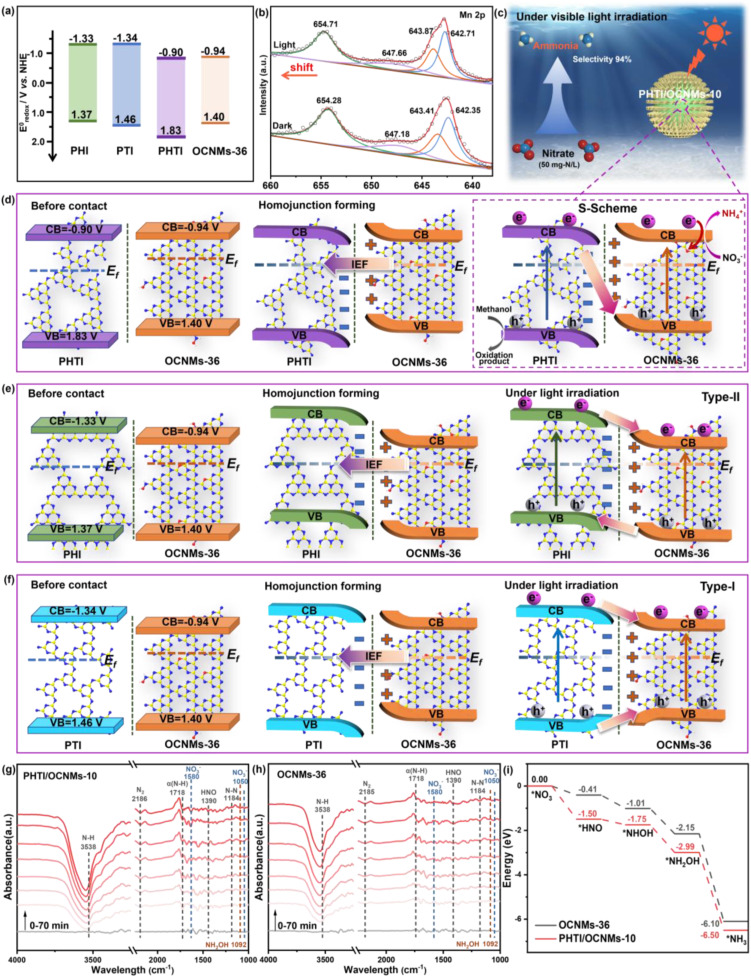
Illustration of the energy band positions of OCNMs-36, PHI, PTI and PHTI (a). *In situ* XPS Mn 2p spectra of metal-coated PHTI/OCNMs-10 (b). Diagrams for the PHTI/OCNMs-10 (c and d), PHI/OCNMs-10 (e) and PTI/OCNMs-10 (f) homojunction before contact, after contact under dark, and under light irradiation for ammonia synthesis. *In situ* DRIFTS of the NO_3_^−^ adsorption and reduction process over PHTI/OCNMs-10 (g) and OCNMs-36 (h). Free-energy diagram for the nitrate adsorption configurations of reaction intermediates on the catalyst surface (i).

Spectroscopic and electrochemical analyses reveal that OCNMs-36 and PHTI possess suitably negative conduction band (CB) positions (−0.94 and −0.90 V *vs.* NHE, respectively; Fig. 4a and S7b–e), enabling thermodynamically feasible nitrate reduction.^42^ Density functional theory (DFT)-calculated work functions of OCNMs-36 (4.72 eV) and PHTI (5.76 eV) indicate a substantial Fermi-level mismatch at their interface (Fig. S12), which drives spontaneous electron transfer from OCNMs-36 to PHTI upon contact. This charge redistribution establishes a strong built-in electric field (IEF) across the homojunction, giving rise to an S-scheme charge transfer configuration (Fig. 4d). Under illumination, low-energy electrons in PHTI recombine with low-energy holes in OCNMs-36, while high-energy electrons are retained in the CB of OCNMs-36 and high-energy holes in the VB of PHTI. This S-scheme PHTI/OCNMs-10 architecture preserves the strongest redox potentials for surface reactions,^43^ in contrast to the type-II and type-I junctions formed in PHI/OCNMs-10 and PTI/OCNMs-10 (Fig. 4e and f), which intrinsically dilute oxidation driving forces. To experimentally elucidate the interfacial S-scheme charge transfer pathway, manganese species were selectively deposited onto PHTI/OCNMs-10 as electron-transfer probes (Fig. S3f), followed by *in situ* XPS measurements under Xe-lamp irradiation. Upon 15 min of light illumination, the Mn 2p core-level spectrum exhibits an evident positive shift in binding energy relative to that in the dark state (Fig. 4b), indicating a decrease in electron density around the Mn cocatalyst sites.^18^ This observation indicates that photogenerated electrons are efficiently extracted from the PHTI component and directionally transferred to OCNMs under the built-in electric field, rather than accumulated on the PHTI surface. It directly verifies the S-scheme charge migration route across the PHTI/OCNMs-10 homojunction, featuring electron migration toward OCNMs-36 and hole retention on PHTI.

To clarify the photocatalytic NO_3_^−^ reduction mechanism on PHTI/OCNMs-10 in comparison with OCNMs-36, *in situ* diffuse reflectance infrared Fourier transform spectroscopy (DRIFTS) was performed. As shown in Fig. 4g, before irradiation over PHTI/OCNMs-10, the characteristic peaks observed at *ca.* 1580 and 1050 cm^−1^ are assigned to the absorbed NO_3_^−^ species.^44^ Upon exposure to visible light for 70 min, the relative intensity of these nitrate-associated features exhibits a pronounced attenuation,^45^ signifying their progressive consumption during the photocatalytic process. Concurrently, new absorption bands emerge at 3538 and 1718 cm^−1^, which can be ascribed to the N–H stretching and *σ*(N–H) vibrations, respectively.^46^ The peaks detected at 2186 and 1184 cm^−1^ are assigned to the N_2_ and N–N stretching vibration modes,^47,48^ while the peak at 1092 cm^−1^ corresponds to the N–O stretching vibration of NH_2_OH.^49^ Furthermore, the emergence of the band at 1390 cm^−1^ is associated with the transient HNO species,^49,50^ highlighting its role as an important reaction intermediate in the overall reduction pathway. In contrast, OCNMs-36 (Fig. 4h) displays substantially weaker signals for the adsorbed NO_3_^−^, intermediates, and products, suggesting inferior nitrate adsorption capability and suppressed photocatalytic reduction activity. This comparison indicates that PHTI incorporation significantly enhances NO_3_^−^ affinity and reaction kinetics.

To further rationalize these experimental observations, density functional theory (DFT)-calculated free energy profiles (Fig. 4i and S13) were analyzed. Compared with pristine OCNMs, PHTI/OCNMs-10 exhibits more favourable *NO_3_ adsorption and significantly reduced energy barriers along the successive hydrogenation pathway (*NO_3_ → *HNO → *NHOH → *NH_2_OH → *NH_3_). The thermodynamically facilitated formation of key intermediates, together with the enhanced stabilization of the final *NH_3_ state, indicates accelerated proton–electron transfer and a more efficient reduction process. Thus, the combined DRIFTS and DFT results demonstrate that PHTI modification optimizes both adsorption and reaction energetics, thereby promoting the selective photocatalytic reduction of NO_3_^−^ to NH_3_. Accordingly, the photocatalytic mechanism over PHTI/OCNMs-10 is governed by an S-scheme-driven redox separation (Fig. 4c and d). Electrons accumulated on oxygen-doped sites of OCNMs-36 selectively reduce NO_3_^−^ through HNO-mediated multielectron pathways, whereas holes localized on PHTI efficiently oxidize methanol.^33,51^ The hierarchical porosity of PHTI/OCNMs-10 further facilitates mass transport and interfacial reactions. The cooperative effects of band alignment, internal electric field, and reaction energetics suppress N_2_-forming side reactions and favor NH_3_ as the dominant product, accounting for the exceptional activity and selectivity of the PHTI/OCNMs-10 homojunction.

### 2.5 The generality of visible-light-driven nitrate reduction

**Fig. 5:**
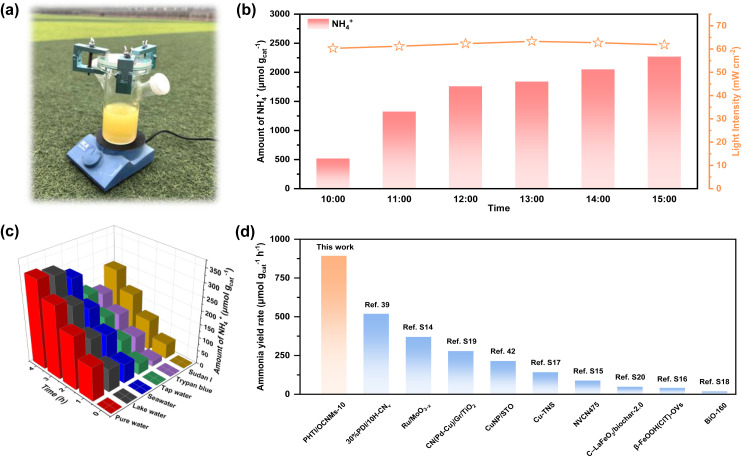
Image of the outdoor test setup equipped with PHTI/OCNMs-10 on 23rd September 2025, at Nanjing Forestry University, Nanjing, China (latitude 32.07°N, longitude 118.81°E) with outdoor temperatures of 22–25 °C (a). Ammonia production rates and incident light intensity on PHTI/OCNMs-10 in outdoor reaction under actual sunlight during the selected period time of 09:00–15:00 for 6 h (b). Photocatalytic nitrate reduction tests in various real water environments over PHTI/OCNMs-10 (c). The comparison of ammonia yield rate from nitrate reduction between PHTI/OCNMs-10 and the reported catalysts (d).

Despite quantitative variations among different systems, a consistent qualitative trend validates the catalytic feasibility toward photocatalytic nitrate reduction. To approach practical application, an ideal configuration should directly exploit natural solar radiation. Accordingly, full-spectrum outdoor photocatalytic nitrate reduction experiments were performed under ambient conditions (Fig. 5a and b). From 10 a.m. to 3 p.m., the increasing solar intensity correlated with a progressive enhancement in ammonia production, reaching a maximum of 2269 µmol g_cat_^−1^. These results highlight the viability of natural sunlight as a dual source of photonic and thermal energy, synergistically enabling selective nitrate conversion. Control experiments using temperature-controlled dark reactions and isothermal visible-light irradiation reveal that photothermal heating accelerates the reaction kinetics, whereas the enhanced activity mainly originates from photocatalytic charge-transfer processes under visible-light irradiation (Fig. S11h).

To assess the real-water applicability of the homojunction catalyst, photocatalytic nitrate reduction was performed in tap, lake, and seawater without hole scavengers. As shown in Fig. 5c, NO_3_^−^-to-NH_4_^+^ conversion efficiencies are lower than in deionized water, primarily due to competitive adsorption of mineral salts on PHTI/OCNMs-10 active sites, which impede proton–catalyst interactions.^16^ Despite this reduction, measurable ammonia production is still achieved after 4 hours of irradiation, yielding 201, 317, and 284 µmol g_cat_^−1^ in tap water, lake water, and seawater, respectively. These results underscore the potential applicability of the homojunction catalyst in diverse water matrices. Notably, seawater, comprising over 96.5% of global water resources, could serve as an abundant and economically viable medium for photocatalytic ammonia synthesis, thereby alleviating pressure on freshwater supplies. To further simulate harsh environmental conditions, two commonly encountered industrial dyes, Trypan blue and Sudan I, were introduced into deionized water at concentrations of 5 mg L^−1^, representative of polluted wastewater. The ammonia yield in these dye-containing solutions declines, attributed to the optical interference and light attenuation caused by the dyes.^52^ Nevertheless, the continued ammonia generation in these conditions demonstrates the resilience and functional adaptability of the PHTI/OCNMs-10 homojunction in turbid or contaminated water systems. As illustrated in Fig. 5d, the ammonia production from nitrate reduction under visible light irradiation and sacrificial reagents ranks among the highest reported, highlighting both its superior photocatalytic efficiency and practical applicability.

## 3. Conclusions

Crystalline-phase-engineered carbon nitride homojunctions provide an effective platform for selective photocatalytic nitrate to ammonia conversion. By integrating PHI, PTI, and PHTI into oxygen-doped carbon nitride microspheres, distinct type-II, type-I, and S-scheme architectures were constructed, enabling direct correlation between band alignment and catalytic performance. The urchin-like S-scheme PHTI/OCNMs-10 exhibits the highest activity and selectivity, achieving an ammonia production rate of 893 µmol g_cat_^−1^ h^−1^ with NH_4_^+^ selectivity exceeding 94%. Mechanistically, a strong built-in electric field arising from the work-function mismatch drives directional S-scheme charge separation, preserving high-energy electrons on OCNMs for nitrate reduction and high-energy holes on PHTI for oxidation. *In situ* XPS, DRIFTS, and DFT analyses further reveal that oxygen-doped sites and the S-scheme configuration synergistically enhance nitrate adsorption, stabilize *HNO-mediated intermediates, and lower the free-energy barriers toward ammonia, thereby suppressing N_2_-forming side reactions. Moreover, the catalyst maintains high catalytic activity under natural sunlight and exhibits robust performance across various real-water environments, highlighting its practical applicability and commercial potential. This work offers a novel strategy for structural regulation of CN-based catalysts to enhance interfacial charge transfer for the advancement of sustainable energy conversion and environmental remediation.

## 4. Experimental section

### 4.1 Materials

All reagents were purchased from commercial suppliers and used without further purification. Dicyandiamide (C_2_H_4_N_4_, 99%) was purchased from Adamas-Beta Co., China. Cyanuric chloride (C_3_Cl_3_N_3_, 99%), sodium (*meta*)periodate (NaIO_4_, 99%) and lithium chloride (LiCl, 98%) were obtained from Shanghai Aladdin Biochemical Technology Co., China. Melamine (C_3_H_6_N_6_, analytical reagent grade) was supplied by Shanghai Lingfeng Chemical Reagent Co., Ltd. Potassium chloride (KCl, 99.8%), methanol (analytical reagent grade), potassium sodium tartrate tetrahydrate, manganese sulfate monohydrate (MnSO_4_·H_2_O, 99%) and potassium nitrate (KNO_3_) were obtained from Sinopharm Chemical Reagent Co., Ltd. Natural seawater (salinity: 32‰, ∼17 000 mg L^−1^ Cl^−^) was collected from the East Sea, Zhejiang Province, China, while real lake water was sampled from Xuanwu Lake in Nanjing, China.

### 4.2 Synthesis of the photocatalysts

PHTI was synthesized *via* a molten salt method.^53^ Melamine (8 g) was calcined at 500 °C for 4 h (12 °C min^−1^, air) to yield bulk CN. CN (1.2 g) was then mixed with KCl (6.6 g) and LiCl (5.4 g), calcined at 550 °C for 4 h (5 °C min^−1^, air), washed with boiling water, and dried at 60 °C to obtain PHTI. PTI was prepared identically, replacing CN with melamine. PHI followed the PHTI procedure but was calcined under N_2_.

The OCNMs were prepared by a solvothermal approach.^33^ Dicyandiamide (1.387 g), cyanuric chloride (2.766 g), and acetonitrile (60 mL) were mixed in air and stirred for 12 h. The solution was sealed in a 100 mL Teflon autoclave and heated at 180 °C for *x* h (*x* = 24, 30, 36, 42, 48). Products were washed with acetonitrile, deionized water, and ethanol, then dried at 80 °C. PHTI/CNMs-*y* (*y* = 5, 10, 15, 20) were prepared using the 36 h solvothermal method with varying PHTI loadings (0.05, 0.1, 0.15, 0.2, and 0.3 g). PHI/OCNMs-10 and PTI/OCNMs-10 composites were also prepared for comparison. The synthesis route is outlined with corresponding colour changes of the prepared catalysts (Fig. S1 and 1j).

### 4.3 Characterization

Morphological features were examined using SEM (Regulus8100, Japan) equipped with an EDX analyzer, and TEM (JEOL 2100F, 200 kV, Japan). Elemental analysis was performed on a PerkinElmer 2400 instrument (USA). Crystal structures were analyzed by powder XRD (Rigaku Ultima IV, Cu Kα, Japan), while surface functional groups were characterized *via* FTIR (Thermo Nicolet-360, USA). Surface chemical states were probed by XPS (Thermo Escalab 250Xi, USA). Optical absorption was recorded using UV-vis DRS (Shimadzu-2600, Japan). Steady-state PL spectra were obtained with a Hitachi F-7000 spectrometer (330 nm excitation, Japan), and time-resolved PL measurements were performed using time-correlated single-photon counting (TCSPC, MT200, Germany) with a 406 nm picosecond diode laser (10 Hz) and a 460/40 nm long-pass filter. Photocurrent responses were measured on a CHI-760E electrochemical workstation (China). *In situ* XPS measurements under light irradiation were carried out on a Kratos Axis Ultra DLD spectrometer using a 300 W Xe lamp as the excitation source.

*In situ* DRIFTS analyses were carried out using a Nicolet iS-50 FTIR spectrometer (Thermo Fisher Scientific) equipped with an MCT detector, a diffuse reflectance accessory, and a high-temperature KBr-windowed reaction chamber. Prior to analysis, the catalyst (50 mg) was immersed in 1.0 M HNO_3_ (25 mL) for 6 h, followed by centrifugation and drying at 80 °C for 12 h. Subsequently, 25 mg of the pre-treated sample was introduced into the DRIFTS cell under a continuous flow of high-purity Ar (99.999%, 50 standard cubic centimeters per minute) to maintain an inert atmosphere. Time-resolved IR spectra were recorded under illumination from a 300 W Xe lamp (CEL-PE300L, CEAULIGHT, Beijing).

### 4.4 Photocatalytic nitrate reduction

The nitration photoreduction reaction was conducted in a 200 mL water-cooled quartz reactor maintained at ambient temperature. The reaction mixture consisted of the photocatalyst (10 mg) dispersed in 50 mL of deionized water containing 10 vol% methanol as a sacrificial agent. Prior to irradiation, Ar was bubbled through the suspension for 30 min to remove dissolved oxygen. The solution was initiated by illumination with a 300 W Xe lamp (≥400 nm). Aliquots (1 mL) were withdrawn at 30 min intervals, filtered to remove residual solids, and analyzed for ammonia content using a colorimetric assay with Nessler's reagent. The labeled ^15^NO_3_^−^ isotope experiments were conducted using a Bruker nuclear magnetic resonance spectrometer (NMR) to confirm the ammonia source. The detailed procedures for the isotope labeling experiment, the determination of by-products, AQE, and density functional theory computational methods are provided in the SI.

## Author contributions

Yiyang Chen: writing – original draft, methodology, investigation, formal analysis, data curation. Yuxiang Zhu: writing – review and editing, methodology, funding acquisition, supervision, conceptualization. Xiang Zhong: investigation, formal analysis. Yuhang Liang: writing – review and editing, methodology. Qiufan Sun: methodology, formal analysis. Sai Xu: writing – review and editing, formal analysis. Jianfeng Yao: writing – review and editing, supervision.

## Conflicts of interest

There are no conflicts to declare.

## Supplementary Material

SC-017-D6SC01863G-s001

## Data Availability

The data that support the findings of this study are available on request from the corresponding author. Supplementary information (SI): experimental and computational methods, structural characterizations, electrochemical studies, comparative photocatalytic performance results, and detailed DFT calculation results. See DOI: https://doi.org/10.1039/d6sc01863g.
