# Supplementary material for: Crystalline–amorphous homojunction engineering in carbon nitride for solar-driven nitrate into ammonia conversion
Source: Chem Sci. 2026 May 29;17(27):13618–28. doi: 10.1039/d6sc01863g (PMC13234772; doi:10.1039/d6sc01863g)
Supplement: SC-017-D6SC01863G-s001 [file SC-017-D6SC01863G-s001.pdf]

Supplementary Information (SI) for Chemical Science.
This journal is © The Royal Society of Chemistry 2026

Supporting Information for

Crystalline-amorphous homojunction engineering in carbon nitride for solar-driven nitrate into ammonia conversion

Supplementary Text

S1. Determination of by-products

Methods and procedures for the determination of by-products:

NO_3^-:

Take 1 mL of the sample solution, add 4 mL of water to dilute it to 5 mL. Then, add 0.1 mL of 1 M hydrochloric acid and 0.01 mL of 0.8 wt% sulfamic acid solution to the above mixture. Let it stand for 15 min, and measure the intensities at 220 nm and 275 nm using a UV-Vis spectrophotometer. The calculation formula is: $A=A_{220}-2A_{275}$.[1]

NO_2^-:

Dissolve 0.5 mg of sulfanilic acid in 90 mL of deionized water, then add 5 mL of glacial acetic acid. After thorough mixing, add 25 mg of ethylenediamine hydrochloride to prepare the chromogenic reagent. Take 1 mL of the reaction solution from the photocatalytic system, add 0.5 mL of the chromogenic reagent, and let it stand for 20 min. Measure the absorbance at 540 nm using a UV-Vis spectrophotometer.[1]

Product selectivity and transformation rates were determined using the following equations:

$$S(\mathrm{N}H_4{}^+) = \frac{[\mathrm{N}H_4{}^+]}{[\mathrm{N}O_3{}^-]_0 - [\mathrm{N}O_3{}^-]_t}$$

(1)

$$S(\mathrm{N}O_2{}^-) = \frac{[\mathrm{N}O_2{}^-]}{[\mathrm{N}O_3{}^-]_0 - [\mathrm{N}O_3{}^-]_t}$$

(2)

$$\mathrm{S(N_2)} = \frac{[\mathrm{N_2}]}{[\mathrm{N}O_3{}^-]_0 - [\mathrm{N}O_3{}^-]_t}$$

(3)

$$T = \frac{[NO_3^-]_0 - [NO_3^-]_t}{[NO_3^-]_0}$$

(4)

S2. AQE calculations

The apparent quantum efficiency (AQE) was determined at λ = 400 nm by quantifying the ammonia production after 4 h of photocatalytic reaction. The AQE was calculated according to the following equation:

$$AQE = \frac{Number\ of\ reacted\ electrons}{Number\ of\ incident\ photons} = \frac{Number\ of\ generated\ NH_3 \times 8}{Number\ of\ incident\ photons} = \frac{M * N_A * 8}{\frac{I \times A \times t}{hv}}$$

Where M represents the amount of NH_3 generation, N_A represents Avogadro's constant, I is the light intensity, A is the light incident area, t is the light irradiation time, h and v are the Planck constant and the incident light frequency, respectively.

S3. Isotope labeling experiment

To confirm the origin of ammonia via isotopic labeling,[2] a series of standard solutions of $^{15}NH_4Cl$ (5, 10, 15, 20, and 25 mmol/L) was prepared. For quantitative analysis by ^1H NMR spectroscopy, 0.5 mL of each standard solution was mixed with 0.4 g of maleic acid (internal standard), 100 μL of 4 M H_2SO_4, and 250 μL of d_6-DMSO. A calibration curve was constructed by plotting the peak area ratio of NH_4^+ to maleic acid against the ammonium concentration, based on their linear correlation. A comparable calibration curve for $^{14}NH_4^+$ was established using standards derived from KNO_3. For evaluation of the photocatalytic ammonia synthesis, following 8 hours of light-driven reaction in either $K^{15}NO_3$ or KNO_3 solution, 0.5 mL of the resulting reaction mixture was subjected to ^1H NMR analysis using the same protocol. The quantification of $^{14}NH_4^+$ or $^{15}NH_4^+$ was carried out using the respective calibration curves.

S4. Computational details

All the density functional theory (DFT) calculations were performed using the Vienna Ab-

initio Simulation Package.[3] The project-augmented wave method was applied to describe the core-valence interaction.[4] The Perdew-Burke-Ernzerhof functional, which uses the generalized gradient approximation, was employed to handle the exchange and correlation potential.[5] A cutoff energy of 450 eV with an energy convergence threshold of 1×10^{-5} eV and a force convergence criterion of 0.02 eV/Å was used. To avoid the interaction between neighboring layers, a 15 Å vacuum layer in the z-direction was set. A 3 × 3 × 1 k-mesh of the Brillouin zone was employed. The DFT-D3 method was also employed to describe the van der Waals interactions.[6] The theoretical work was supported by computational resources provided by the Australian Government through Gadi and Pawsey under the National Computational Merit Allocation Scheme and was accessed through the SIH HPC Allocation Scheme [LE190100021]. To get the adsorption models, the optimization was carried out with the mixed Gaussian plane wave scheme using the CP2K package.[7-9] Then, the Perdew-Burke-Ernzerhof (PBE) exchange-correlation functional,[5] and the DZVPMOLOPT-SR basis set with Goedecker-Teter-Hutter pseudo potentials were used.[10] During the calculation, the Grimme D3 correction with zero damping was applied to account for the dispersion interactions,[11] as well as the plane wave cutoff energy and relative cutoff were 400 Ry and 55 Ry, respectively. The electrostatic potential of the system was calculated using the DMol3 package.[12] Density functional theory calculations were performed employing the PBE exchange-correlation function within the generalized gradient approximation. Thermodynamic corrections to the electronic energies were obtained using the Shermo program.[13]

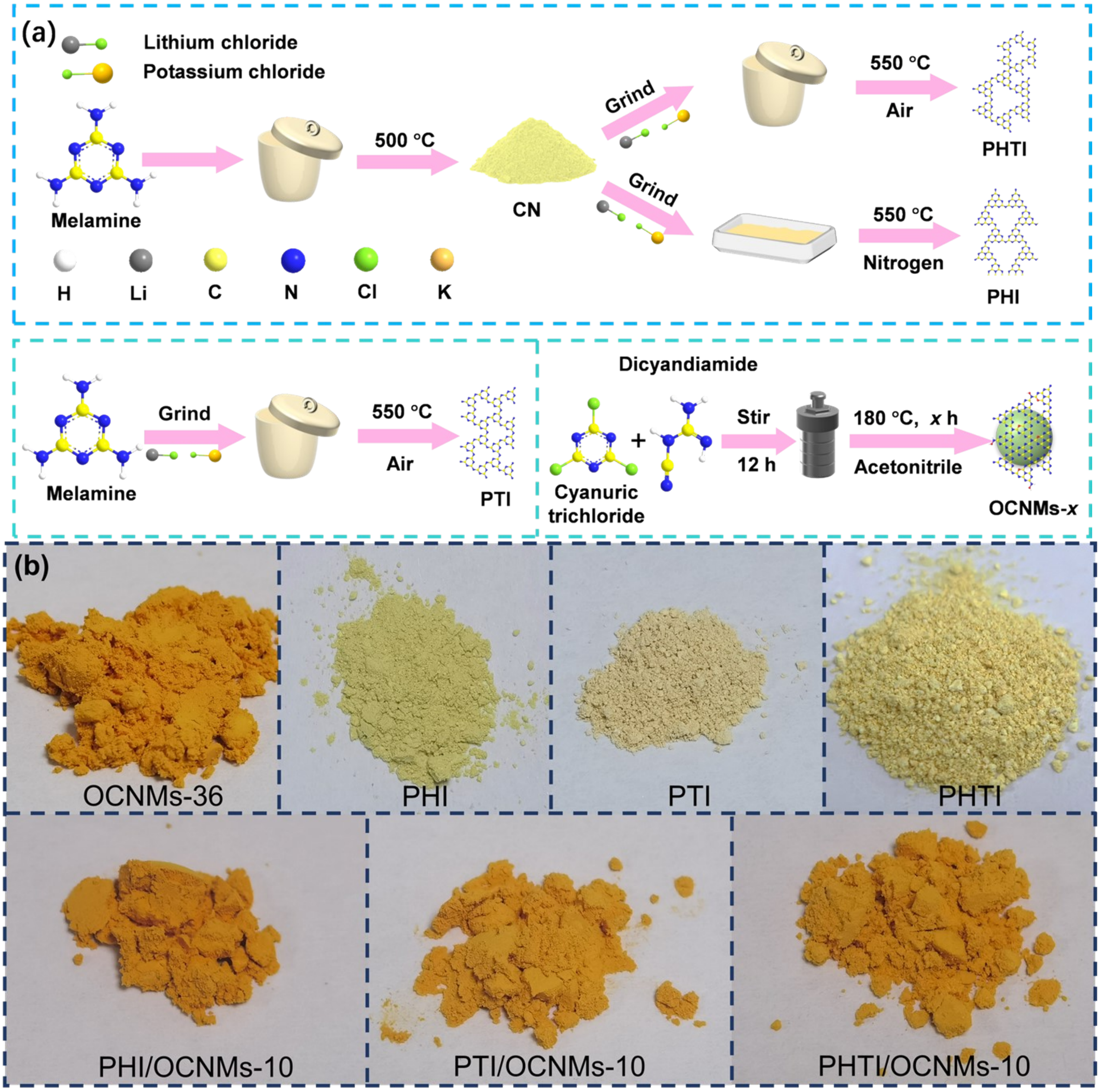

Figure S1 The illustration of the preparation process of PHI, PTI, PHTI and OCNMs (a). The appearance of the synthesized photocatalysts (b).

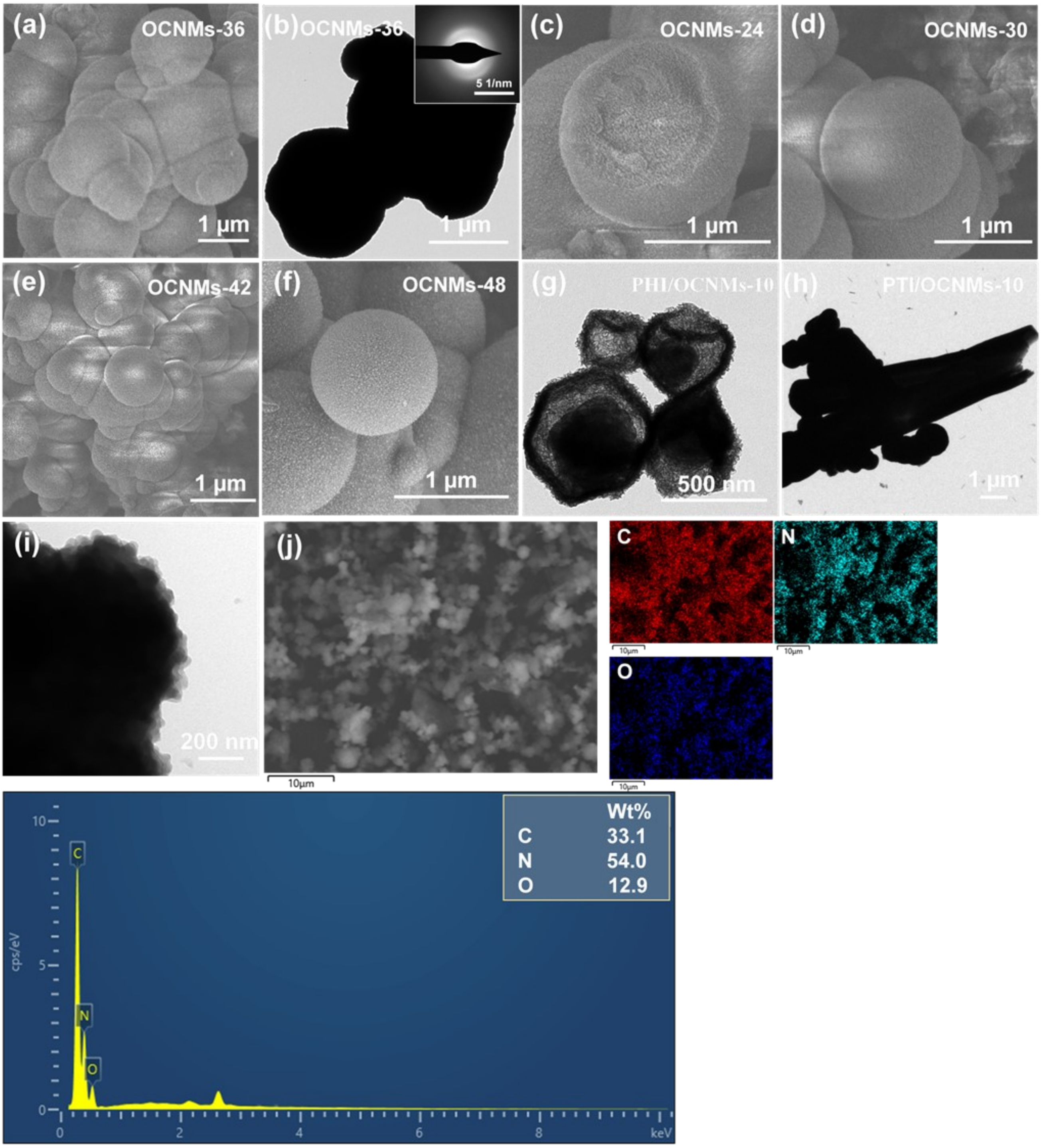

Figure S2 SEM images of OCNMs with different solvothermal durations (24, 30, 36, 42 and 48 h) (a-e). TEM images of OCNMs (f), PHI/OCNMs-10 (g), PTI/OCNMs-10 (h) and TEM PHTI/OCNMs-10 after reaction (i). SEM-EDX mapping of PHTI/OCNMs-10 (j).

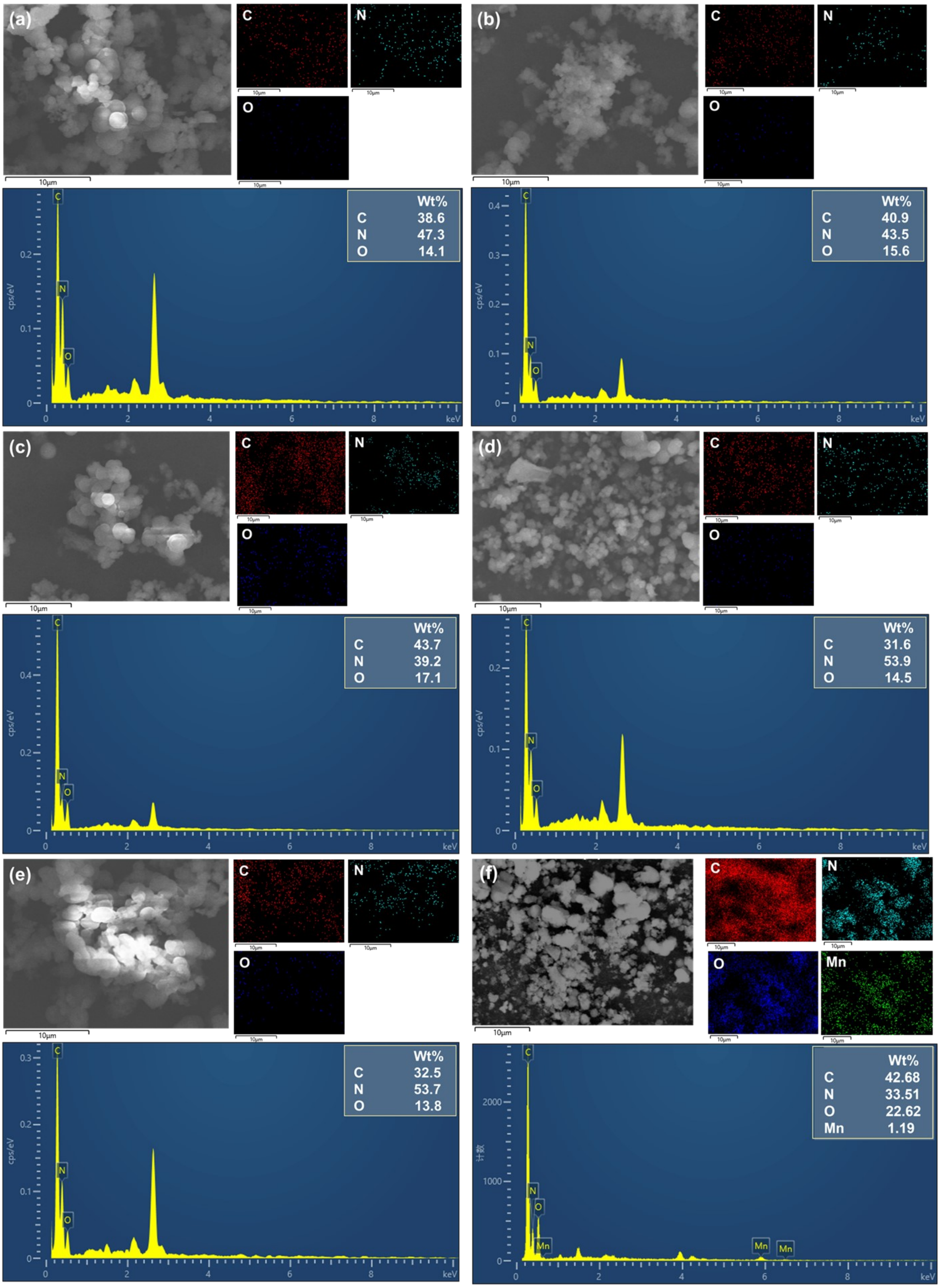

Figure S3 SEM-EDX mapping of OCNMs-24 (a), OCNMs-30 (b), OCNMs-36 (c), OCNMs-42 (d), OCNMs-48 (e) and metal loaded PHTI/OCNMs-36 (f).

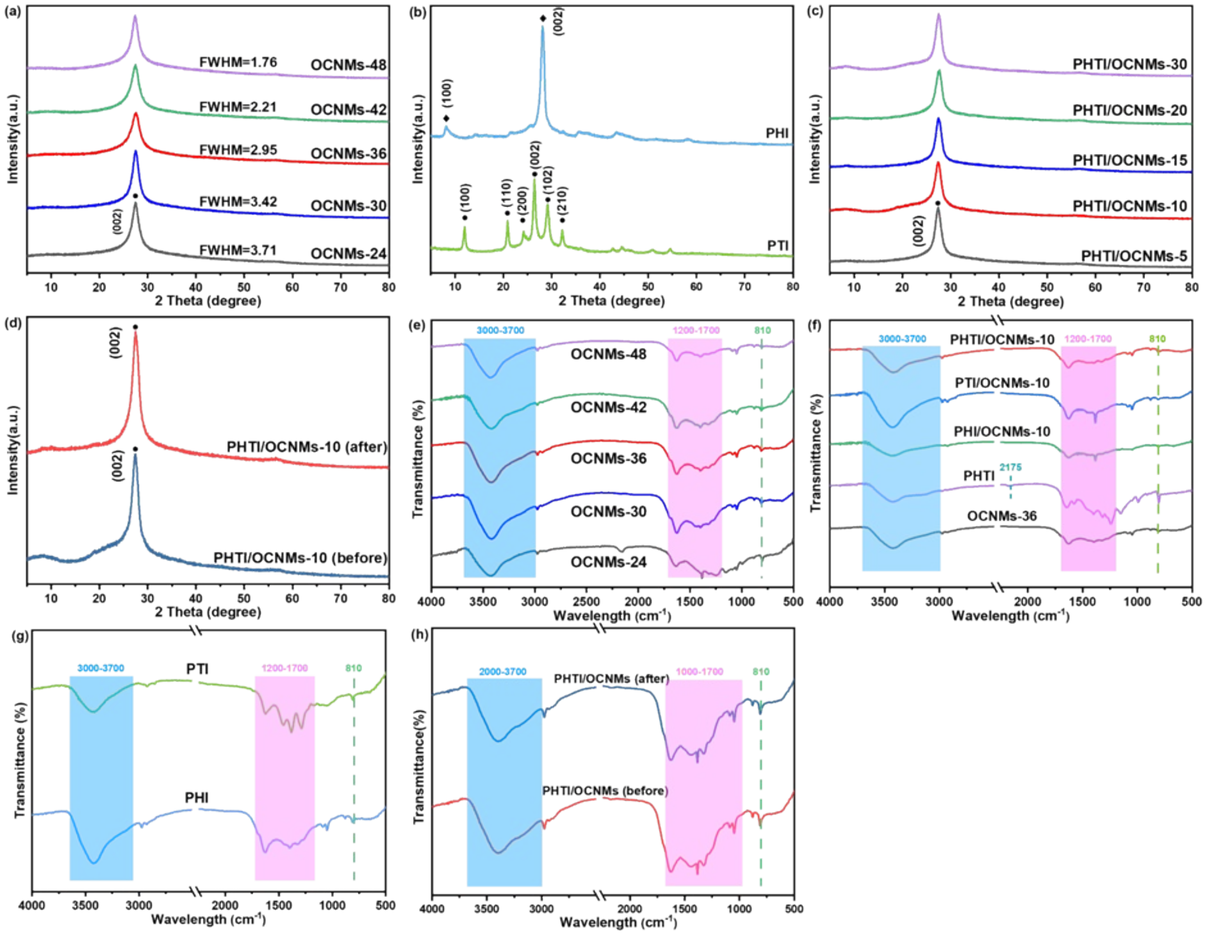

Figure S4 XRD patterns (a) and FTIR spectra (e) of OCNMs with different solvothermal durations (24, 30, 36, 42 and 48 h). XRD patterns of PHI, PTI (b) and PHTI/OCNMs-10 with different loading content (c). XRD patterns (d) and FTIR spectra (h) of PHTI/OCNMs-10 before and after the photocatalytic reactions. FTIR spectra of the OCNMs-36, PHTI, PHI/OCNMs-10, PTI/OCNMs-10, PHTI/OCNMs-10 (f), PHI and PTI (g) catalysts.

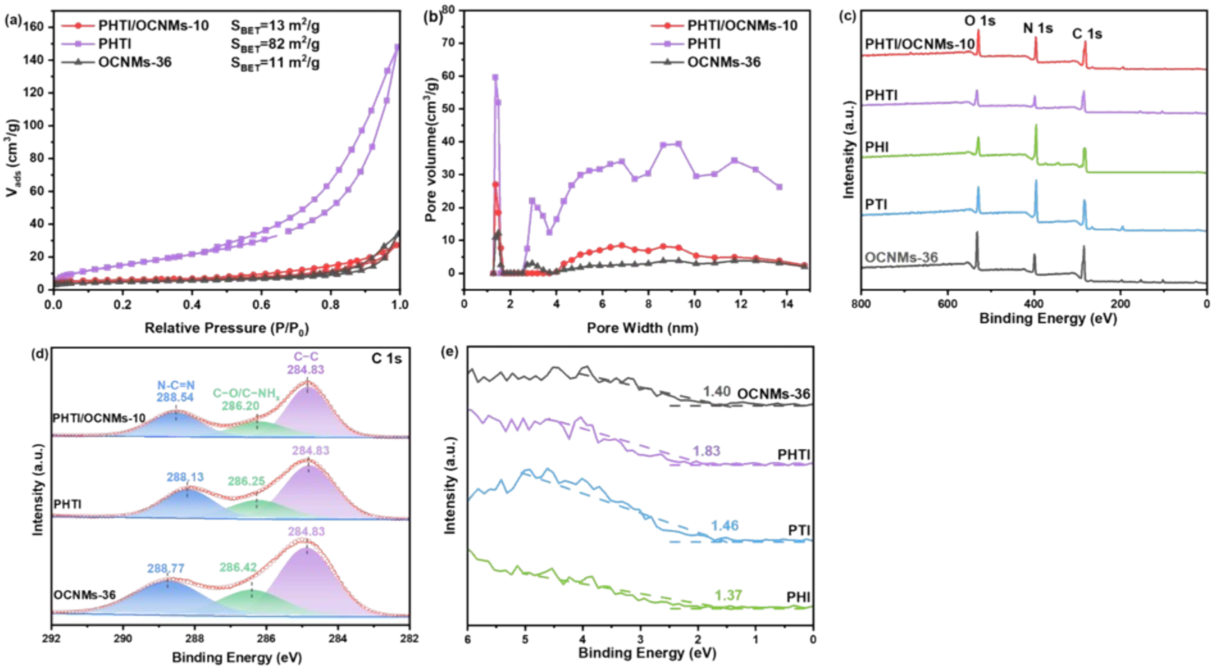

Figure S5 The N_2 adsorption-desorption isotherms (a) and pore size distribution (b) of OCNMs-36 and PHTI/OCNMs-10. Full XPS spectra of OCNMs-36, PHI, PTI, PHTI, and PHTI/OCNMs-10 (c). High-resolution C 1s XPS spectra of PHTI, OCNMs-36 and PHTI/OCNMs-10 (d). The VB XPS spectra (e) of PHI, PTI, PHTI and OCNMs-36.

Table S1 The element contents of the various catalysts

Catalysts	C (wt%)	N (wt%)	O (wt%)	H (wt%)
OCNMs-36	26.3	43.9	26.0	3.7
PHI/OCNMs-10	26.8	45.1	24.4	3.7
PTI/OCNMs-10	25.4	46.7	24.0	4.0
PHTI/OCNMs-10	26.7	43.2	27.7	2.4

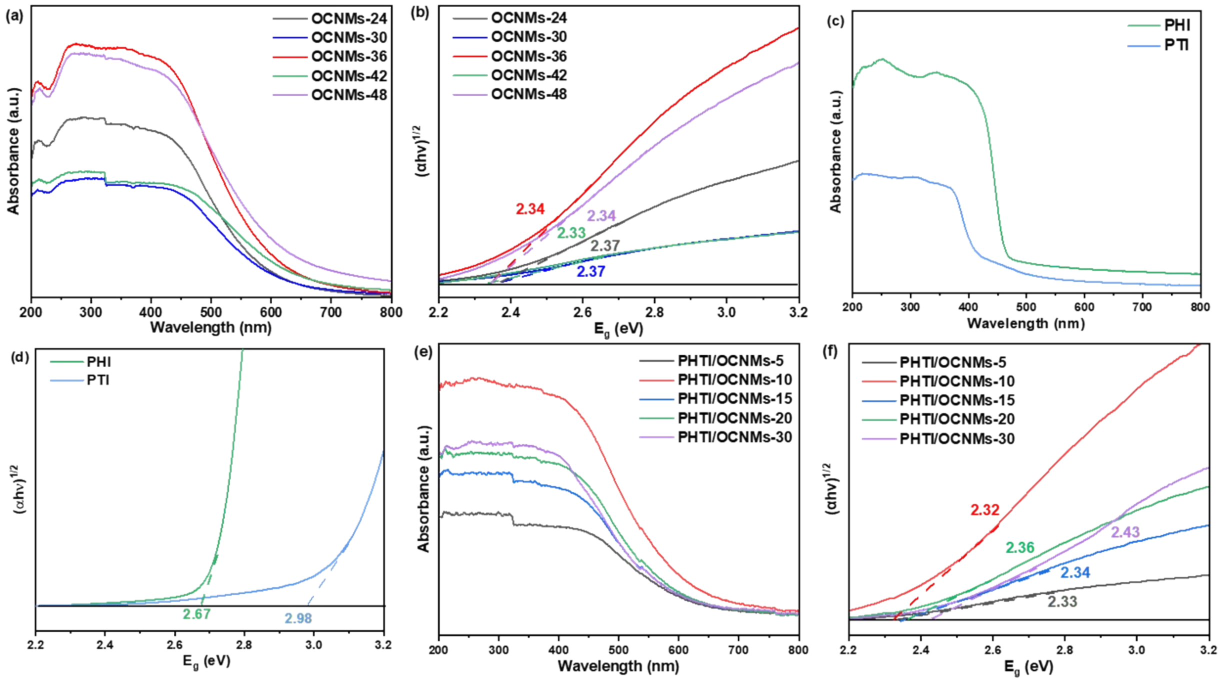

Figure S6 UV-vis DRS spectra of OCNMs-*x* (a), PHI, PTI (c) and PHTI/OCNMs-*y* (e) catalysts, and their corresponding Kubelka-Munk plots (b, d, f).

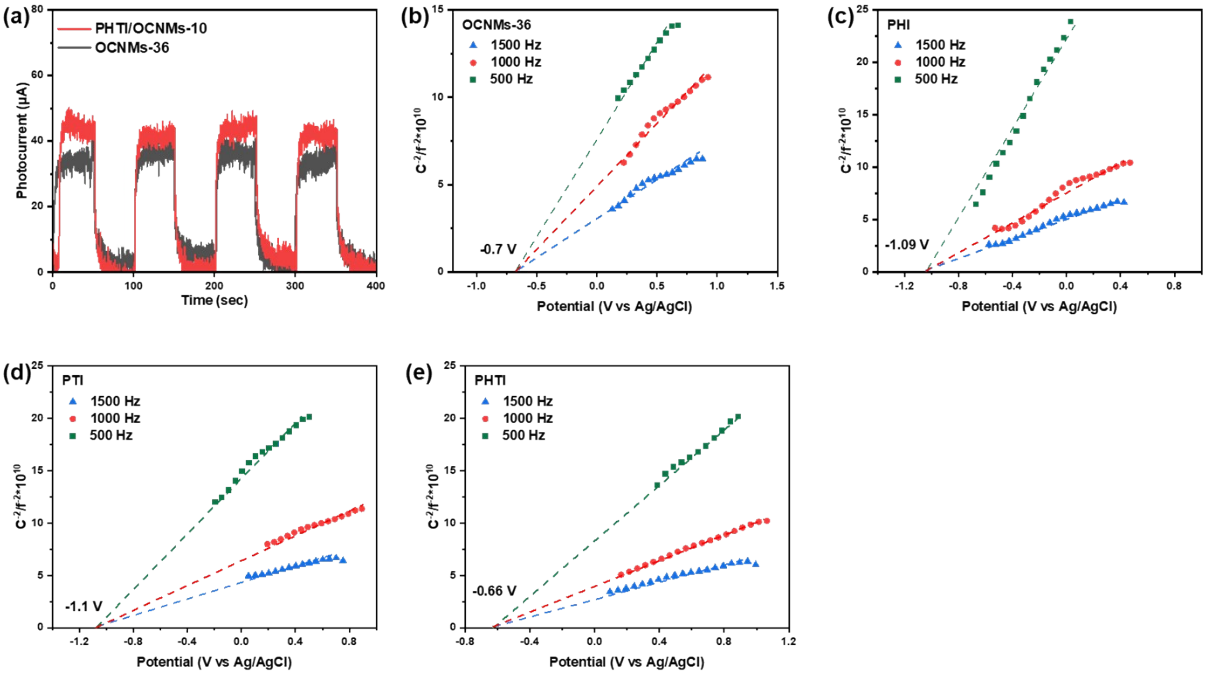

Figure S7 Transient photocurrent responses of OCNMs-36 and PHTI/OCNMs-10 (a). The Mott-Schottky plots of OCNMs-36, PHI, PTI and PHTI (b-e).

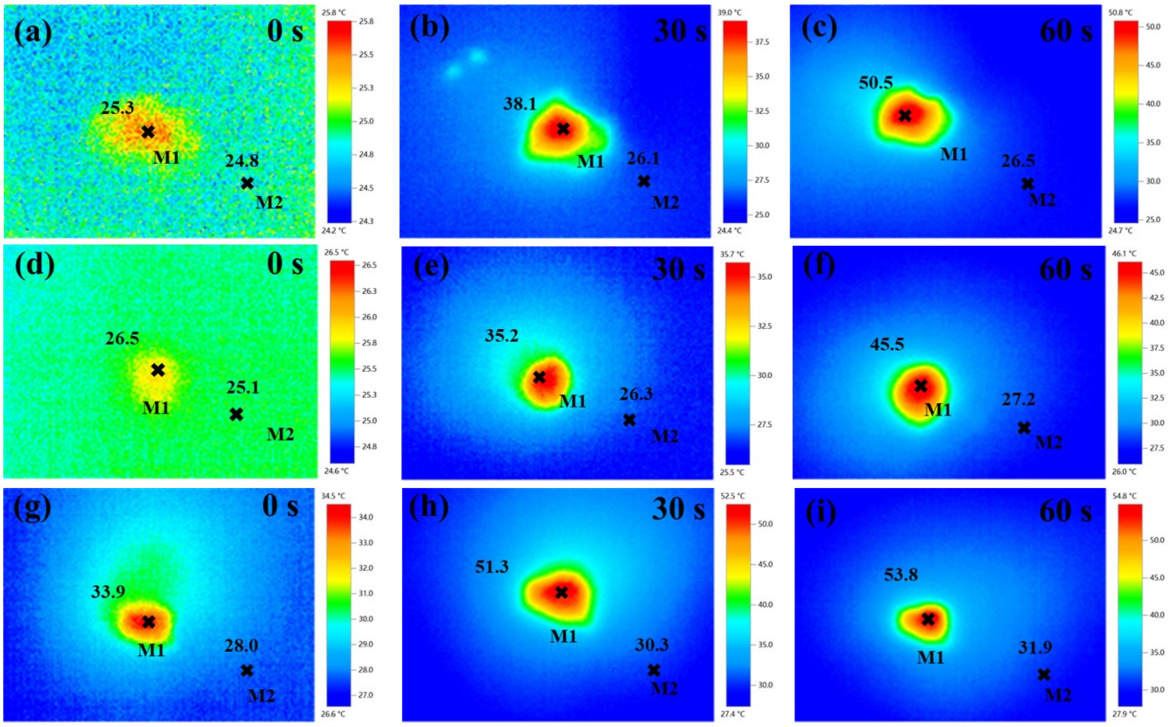

Figure S8 Time-dependent infrared images of OCNMs-36 (a-c), PHTI (d-f), PHTI/OCNMs-10 (g-i).

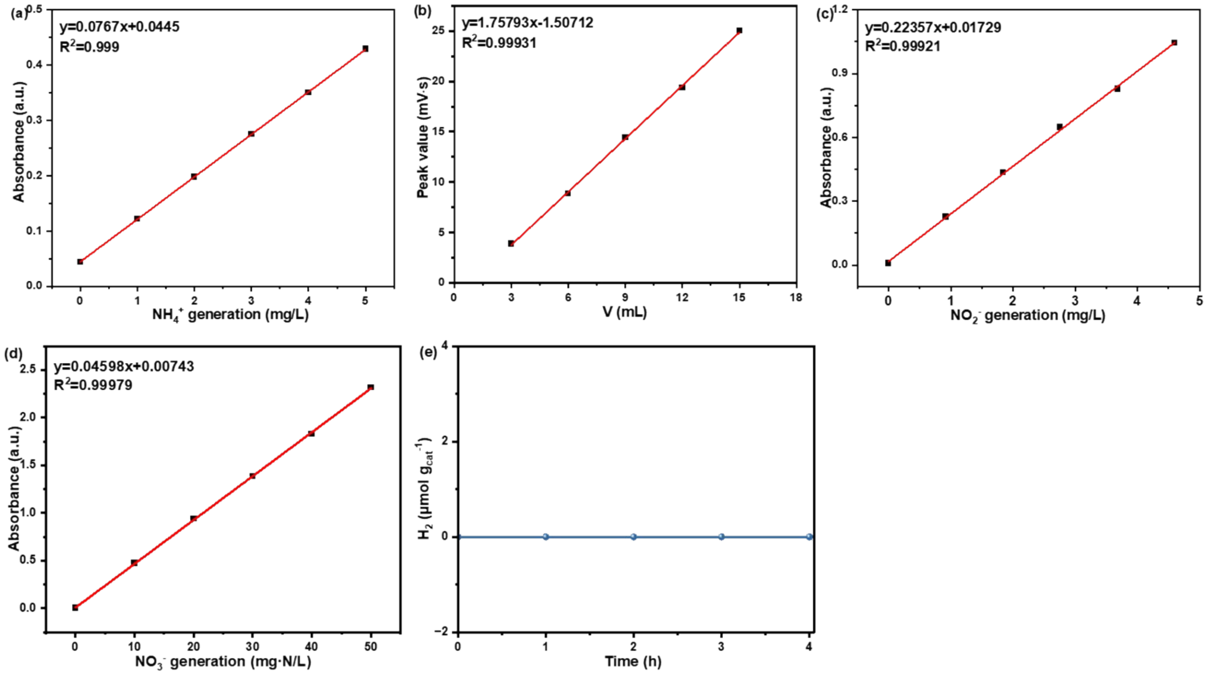

Figure S9 Calibration curves for ammonia by Nessler's reagent (a), N_2 (b), NO_2^- (c) and NO_3^- (d). The content of the produced H_2 during the photocatalytic nitrate reduction process (e).

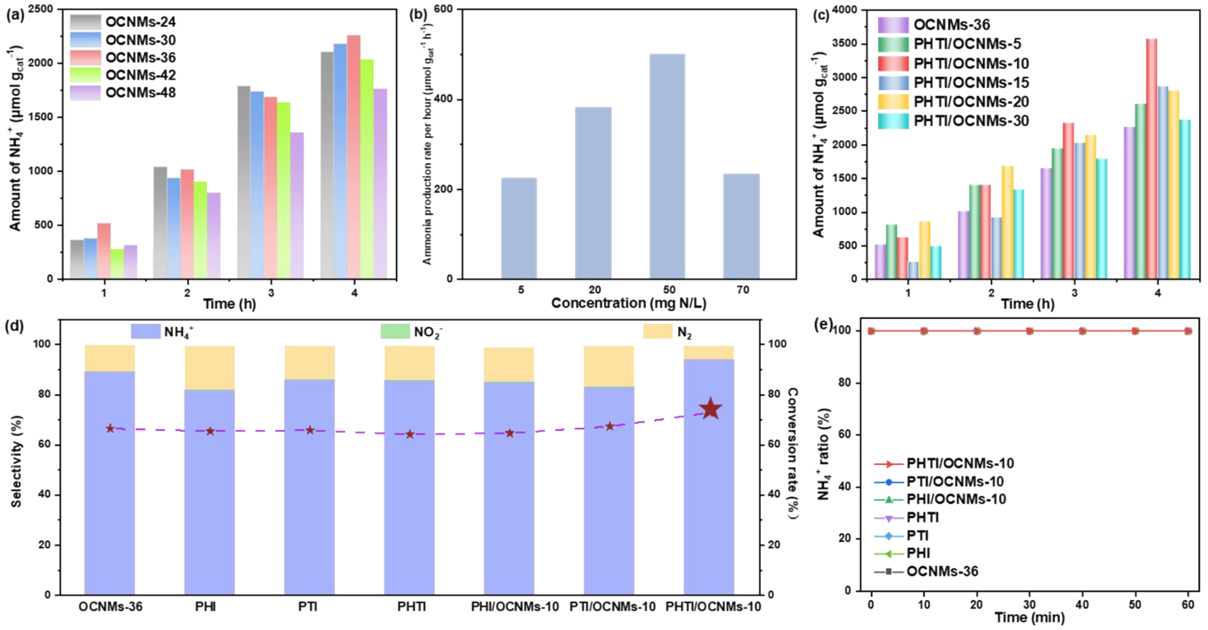

Figure S10 Photocatalytic nitrate reduction tests of OCNMs-*x* (a). Ammonia production rate of different concentrations of nitrate for two hours (b). Photocatalytic nitrate reduction tests of PHTI/OCNMs-*y* (c). Selectivity of OCNMs-36, PHI, PTI, PHTI, PHI/OCNMs-10, PTI/OCNMs-10 and PHTI/OCNMs-10 (d). Time-resolved adsorption experiments for OCNMs-36, PHI, PTI, PHTI, PHI/OCNMs-10, PTI/OCNMs-10 and PHTI/OCNMs-10 in NH_4^+ solution (e).

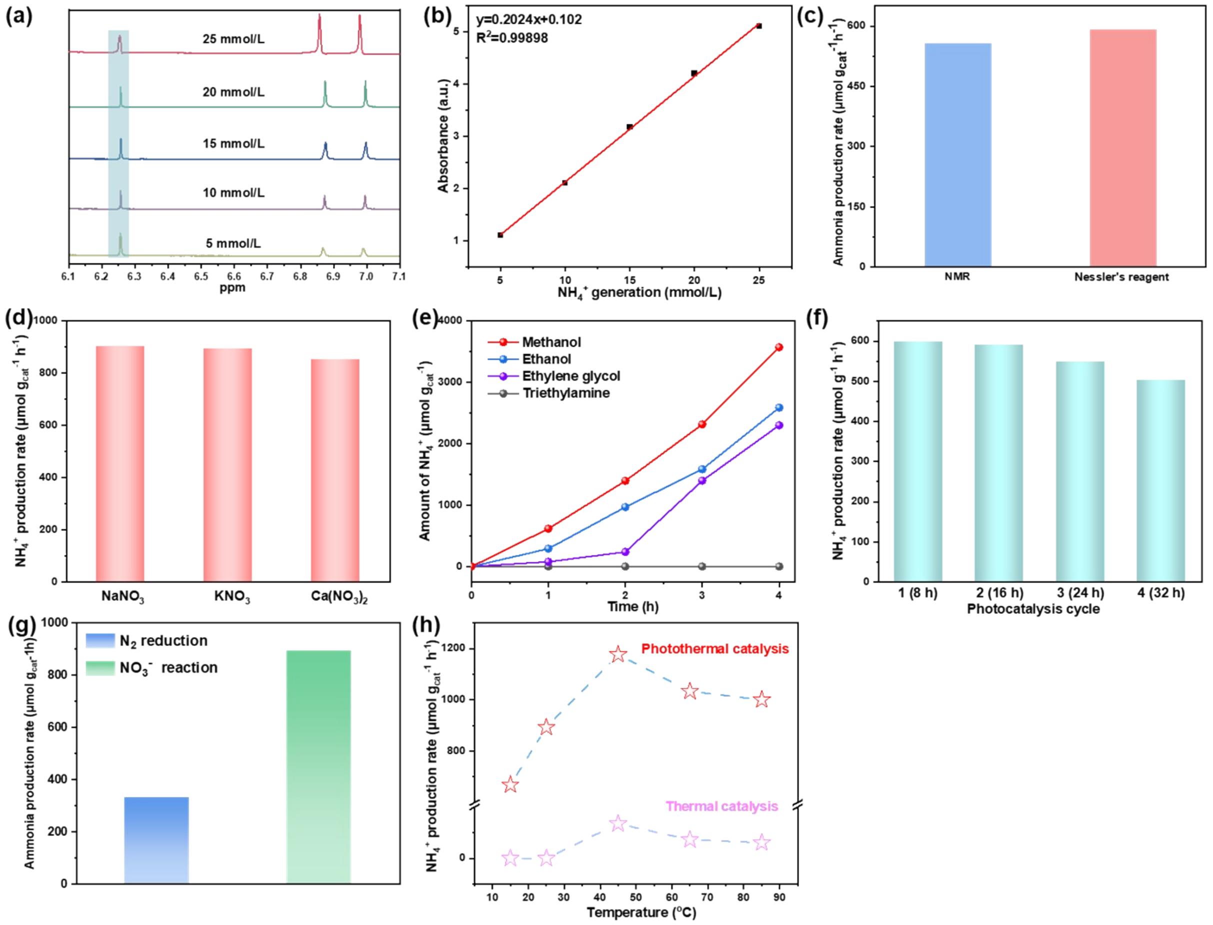

Figure S11 ^{1}H NMR spectra of $^{15}NH_4^+$ at concentrations ranging from 5-25 mmol/L (a), along with the corresponding calibration curves for $^{15}NH_4^+$ (b). The comparison of the generated ammonia amount over PHTI/OCNMs-10 tested by NMR and indophenol and Nessler's methods (c). The photocatalytic activity over various nitrate salts (d) and sacrificial reagents (e). The long-term stability test of PHTI/OCNMs-10 (f). The comparison of photocatalytic ammonia synthesis for N_2 and NO_3^- reduction, respectively (g). The temperature-controlled dark reactions and isothermal visible light-assisted catalytic process over PHTI/OCNMs-10 (h).

The ammonia yield under photothermal conditions was consistently higher than that under purely thermal conditions over the temperature range of 15-85 °C, highlighting the dominant role of photocatalysis (**Figure S11h**). The NH_3 production increases with temperature and reaches a maximum at 45 °C, owing to enhanced charge-transfer kinetics and accelerated

surface reaction dynamics. However, further increasing the temperature to 65-85 °C leads to a gradual decline in NH_3 yield, which could be associated with accelerated electron-hole recombination and reduced light-utilization efficiency at elevated temperatures.[14, 15] Notably, no detectable gaseous NH_3, H_2, N_2, and only trace nitrite were observed (65-85 °C), excluding the influence of side reactions.

Table S2 Comparison of the reported photocatalysts for the photocatalytic nitrate reduction

Photocatalyst	Mass of photocatalyst and reaction solution	Light source	Ammonia yield (μmol g_{cat}^{-1} h^{-1})	AQE (%) Wavelength	Ammonis selectivity	By-products	Ref. (Year)
PHTI/OCNMs-10	10 mg, 45 mL KNO_3 (50 mg N/L) 5 mL methanol	300 W Xe lamp (λ > 400 nm)	893<br>4 h	6.3%<br>400 nm	94%	NO_2^-, N_2, N_2H_4	This work
30%PDI/10H-CN_v	10 mg, 100 mL KNO_3 (75.3 mg N/L)<br>10 wt% methanol	300 W xenon lamp	519<br>1 h	0.6%<br>475 nm	/	/	[16]<br>2025
CuNP/STO	10mg, 150 mL 75.3 mg N/L NO_3^-<br>5 mL methanol	300 W Xe lamp<br>Full spectrum	214<br>4 h	/	84.1%	H_2, N_2	[17]<br>2024
Ru/MoO_{3-x}	20 mg, 100 mL of $NaNO_3$ solution (4.52 mg N/L)	300 W xenon lamp	370<br>4 h	/	/	/	[2]<br>2024
NVCN475	100 mg, 150 mL 2.89 mg N/L of NO_3^-<br>0.5wt% of formic acid	300 W Xe lamp (λ > 420 nm)	88<br>2 h	/	97%	N_2, H_2	[18]<br>2024
β-FeOOH(Cl_T)-OVs	50 mg, 25 mL<br>140.1 mg N/L KNO_3	300 W Xe lamp (λ >400 nm)	41<br>5 h	/	98%	/	[19]<br>2024
Cu-TNS	100 mg, 150 mL KNO_3 ,2.89 mg N/L)<br>HCOOH (0.5 wt%, 15 mmol)	300 W Xe lamp<br>Full spectrum	142<br>24 h	11.7%	98%	/	[20]<br>2023
BiO-160	20 mg, 100 mL	300 W Xe lamp	18<br>4 h	/	/	N_2	[21]<br>2023
CN(Pd-Cu)/Gr/TiO_2	10 mg, 50 mL of NO_3^-	300 W xenon lamp<br>（$\lambda \geq$ 400 nm）	278<br>4 h	/		NO_2^-	[22]<br>2023
C-doped $LaFeO_3$/biochar-2.0	50 mg, 100 mL KNO_3 (70.1 mg N/L)<br>HCOOH	300 W Xe lamp (λ > 420 nm)	48<br>2 h	/	98%	/	[23]<br>2022

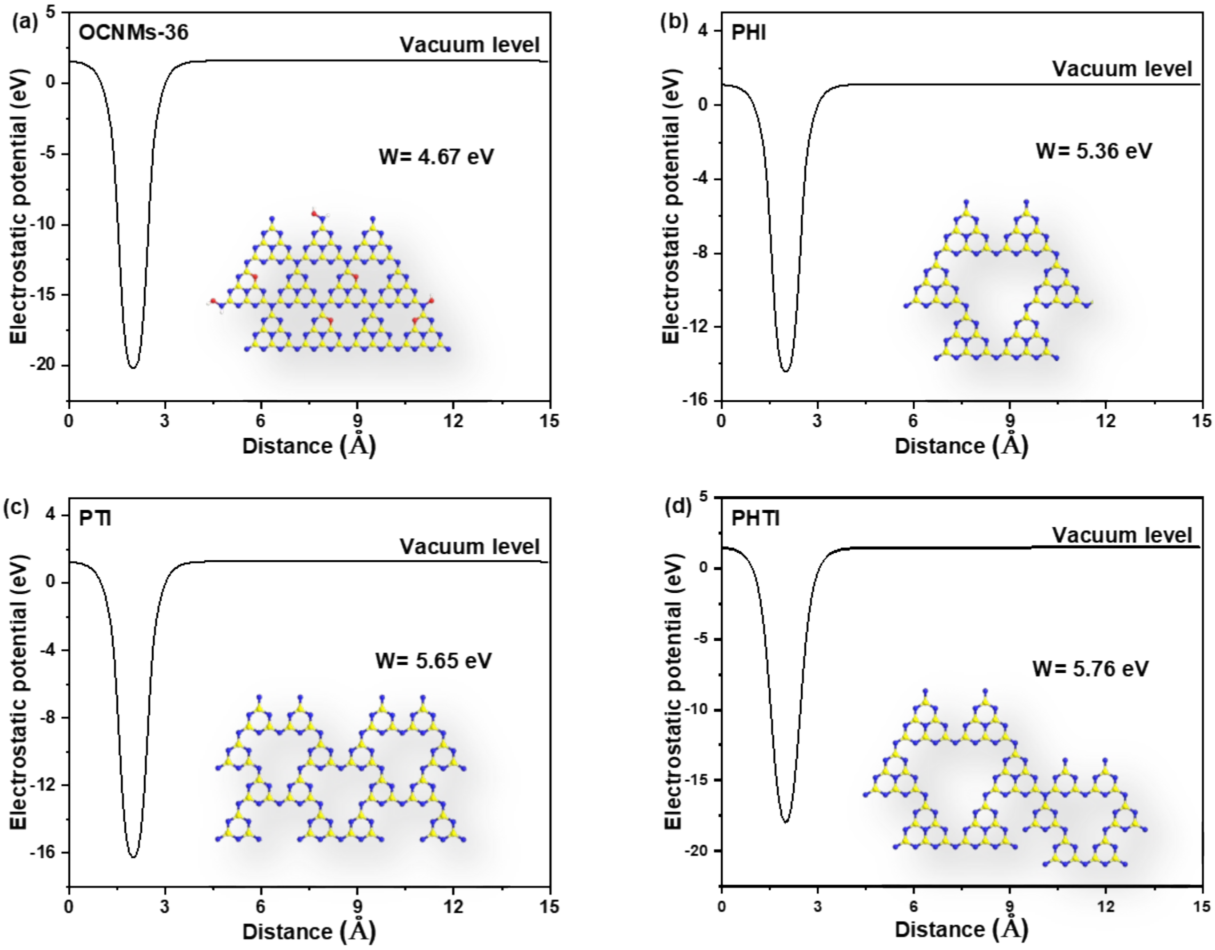

Figure S12 The calculated work functions of OCNMs-36, PHI, PTI and PHTI (a-d).

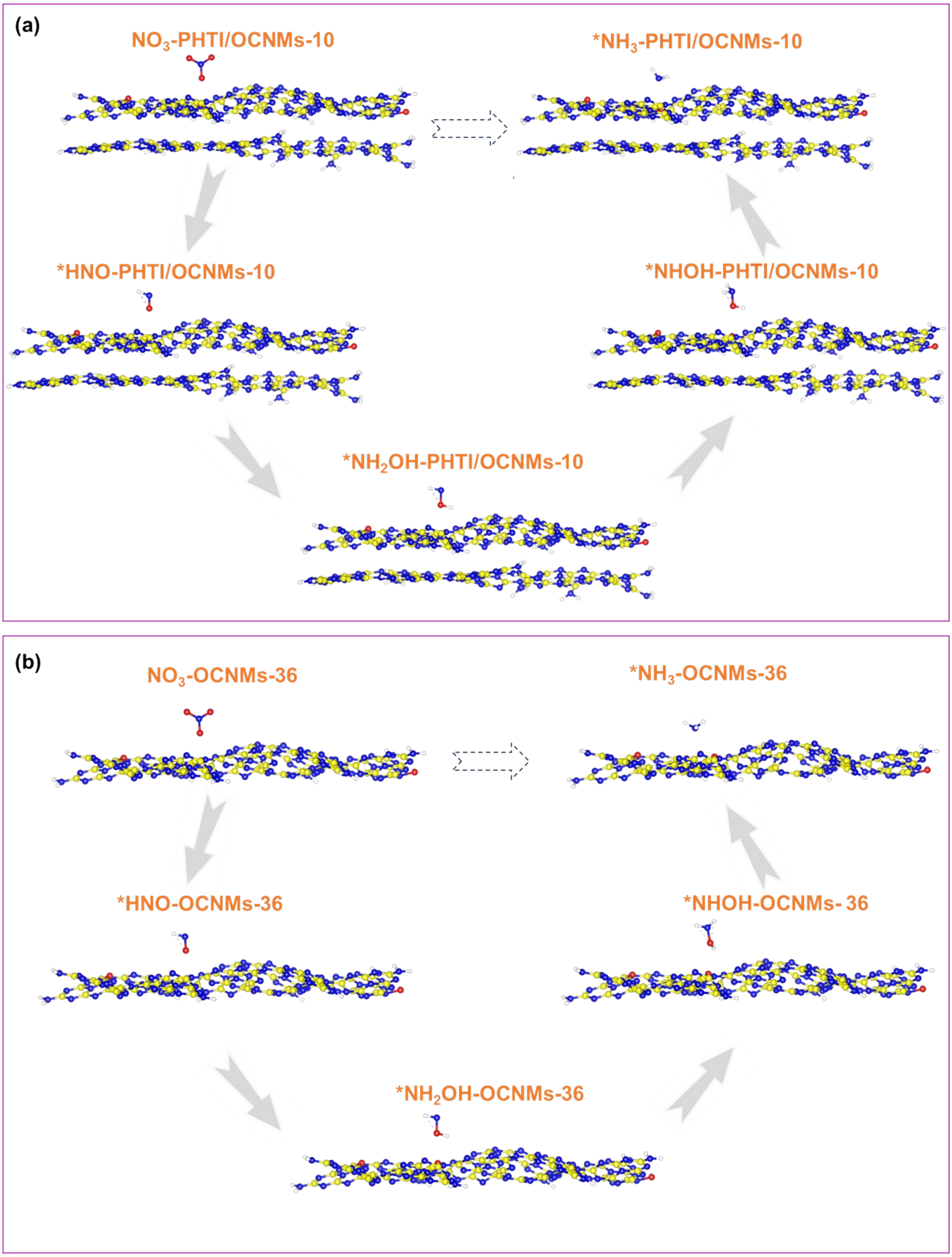

Figure S13 Adsorption models of $^*NO_3$, *HNO, *NHOH, $^*NH_2OH$ and on PHTI/OCNMs-10 (a) and on OCNMs-36 (b).

Reference

1 X. Deng, D. Liu, M. Yuan, Y. Li, H. Yang, C. Wang, R. Wang and X. Yang, *Inorg. Chem.*, 2025, **64**, 2294-2302.

2 L. Zhang, R. Li, L. Guo, L. Cui, X. Zhang, Y. Wang, Y. Wang, X. Jian, X. Gao, C. Fan, J. Wang and J. Liu, *ACS Catal.*, 2024, **14**, 5696-5709.

3 G. Kresse and J. Furthmüller, *Comput. Mater. Sci.*, 1996, **6**, 15-50.

4 G. Kresse and D. Joubert, *Phys. Rev. B*, 1999, **59**, 1758-1775.

5 J. P. Perdew, K. Burke and M. Ernzerhof, Phys. *Rev. Lett.*, 1996, **77**, 3865-3868.

6 M. Dion, H. Rydberg, E. Schröder, D. C. Langreth and B. I. Lundqvist, *Phys. Rev. Lett.*, 2004, **92**, 246401.

7 J. Hutter, M. Iannuzzi, F. Schiffmann and J. VandeVondele, *WIREs Comput. Mol. Sci.*, 2014, **4**, 15-25.

8 J. VandeVondele, M. Krack, F. Mohamed, M. Parrinello, T. Chassaing and J. Hutter, *Comput. Phys. Commun.*, 2005, **167,** 103-128.

9 J. VandeVondele and J. Hutter, *J. Chem. Phys.*, 2007, **127**, 114105.

10 S. Goedecker, M. Teter and J. Hutter, *Phys. Rev. B,* 1996, **54**, 1703-1710.

11 S. Grimme, J. Antony, S. Ehrlich and H. Krieg, *J. Chem. Phys.*, 2010, **132**, 154104.

12 M. J. Rutter, *Comput. Phys. Commun.*, 2018, **225**, 174-179.

13 T. Lu and Q. Chen, *Comput. Theor. Chem.*, 2021, **1200**, 113249.

14 J. He, W. H. Fang and R. Long, *J. Mater. Chem. A*, 2020, **8**, 607-615.

15 Y. Zhou, Y. Feng, H. Xie, J. Lu, D. Ding and S. Rong, *Appl. Catal. B: Environ.*, 2023, **331**, 122668.

16 D. Cui, X. Yang, Y. Liu, M. Li, C. Wang and F. Li, *Sep. Purif. Technol.*, 2025, **356**, 130035.

17 S. O. Lee, G. Youn, D. Lee, Y. Yun, S. K. Lakhera and K. Yong, *Ceram. Int.*, 2025, **51**, 26491-26498.

18 I. Hong, H. S. Moon, B. J. Park, Y. A. Chen, Y. P. Chang, B. Song, D. Lee, Y. Yun, Y. J. Hsu, J. W. Han and K. Yong, *Chem. Eng. J.*, 2024, **484**, 149506.

19 Y. Shiraishi, S. Akiyama, W. Hiramatsu, K. Adachi, S. Ichikawa and T. Hirai, *JACS Au*, 2024, **4**, 1863-1874.

20 H. S. Moon, B. Song, J. Jeon, T. H. Lai, Y. P. Chang, Y. D. Lin, J. K. Park, Y. G. Lin, Y. J. Hsu, H. Shin, Y. Yun and K. Yong, *Appl. Catal. B: Environ.*, 2023, **339**, 123185.

21 D. Hao, Y. Wei, L. Mao, X. Bai, Y. Liu, B. Xu, W. Wei and B. J. Ni, *J. Clean. Prod.*, 2022, **331**, 129975.

22 H. Li, S. Xue, F. Cao, C. Gao, Q. Wei, R. Li, A. Zhou, S. Wang and X. Yue, *Chemosphere*, 2023, **325**, 138336.

23 W. Liu, X. Li, X. Chu, S. Zuo, B. Gao, C. Yao, Z. Li and Y. Chen, *Chemosphere*, 2022, **294**, 133763.
